# Inferring neural circuit structure from datasets of heterogeneous tuning curves

**DOI:** 10.1371/journal.pcbi.1006816

**Published:** 2019-04-19

**Authors:** Takafumi Arakaki, G. Barello, Yashar Ahmadian

**Affiliations:** 1 Institute of Neuroscience, University of Oregon, Eugene, Oregon, USA; 2 Departments of Biology and Mathematics, University of Oregon, Eugene, Oregon, USA; Ghent University, BELGIUM

## Abstract

Tuning curves characterizing the response selectivities of biological neurons can exhibit large degrees of irregularity and diversity across neurons. Theoretical network models that feature heterogeneous cell populations or partially random connectivity also give rise to diverse tuning curves. Empirical tuning curve distributions can thus be utilized to make model-based inferences about the statistics of single-cell parameters and network connectivity. However, a general framework for such an inference or fitting procedure is lacking. We address this problem by proposing to view mechanistic network models as implicit generative models whose parameters can be optimized to fit the distribution of experimentally measured tuning curves. A major obstacle for fitting such models is that their likelihood function is not explicitly available or is highly intractable. Recent advances in machine learning provide ways for fitting implicit generative models without the need to evaluate the likelihood and its gradient. Generative Adversarial Networks (GANs) provide one such framework which has been successful in traditional machine learning tasks. We apply this approach in two separate experiments, showing how GANs can be used to fit commonly used mechanistic circuit models in theoretical neuroscience to datasets of tuning curves. This fitting procedure avoids the computationally expensive step of inferring latent variables, such as the biophysical parameters of, or synaptic connections between, particular recorded cells. Instead, it directly learns generalizable model parameters characterizing the network’s *statistical structure* such as the statistics of strength and spatial range of connections between different cell types. Another strength of this approach is that it fits the joint high-dimensional distribution of tuning curves, instead of matching a few summary statistics picked *a priori* by the user, resulting in a more accurate inference of circuit properties. More generally, this framework opens the door to direct model-based inference of circuit structure from data beyond single-cell tuning curves, such as simultaneous population recordings.

## Author summary

Neurons in the brain respond selectively to some stimuli or for some motor outputs, but not others. Even within a local brain network, neurons exhibit great diversity in their selectivity patterns. Recently, theorists have highlighted the computational importance of diverse neural selectivity. While many mechanistic circuit models are highly stylized and do not capture such diversity, models that feature biologically realistic heterogeneity in their structure do generate responses with diverse selectivities. However, traditionally only the average pattern of selectivity is matched between model and experimental data, and the distribution around the mean is ignored. Here, we provide a hitherto lacking methodology that exploits the full empirical and model distributions of response selectivites, in order to infer various structural circuit properties, such as the statistics of strength and spatial range of connections between different cell types. By applying this method to fit two circuit models from theoretical neuroscience to experimental or simulated data, we show that the proposed method can accurately and robustly infer circuit structure, and optimize a model to match the full range of observed response selectivities. Beyond neuroscience applications, the proposed framework can potentially serve to infer the structure of other biological networks from empirical functional data.

## Introduction

Neural responses in many brain areas are tuned to external parameters such as stimulus- or movement-related features. Tuning curves characterize the dependence of neural responses on such parameters, and are a key descriptive tool in neuroscience. Experimentally measured tuning curves often exhibit a rich and bewildering diversity across neurons in the same brain area, which complicates simple understanding (*e*.*g*., see [1–4]). This complexity has given rise to a tendency towards biased selections of minorities of cells which exhibit pure selectivites, and have orderly and easily interpretable tuning curves. As a result the biological richness and diversity of tuning curves in the full neural population is often artificially reduced or ignored. On the theoretical side too, many network models feature homogeneous populations of cells with the same cellular parameters and with regular synaptic connectivity patterns. Neural tuning curves in such models will naturally be regular and have identical shapes.

New theoretical advances, however, have highlighted the computational importance of diverse tuning and mixed selectivity, as observed in biological systems [3, 5]. Furthermore, diversity and heterogeneity can be produced in mechanistic network models which either include cell populations with heterogeneous single-cell parameters (see *e*.*g*., Ref. [2]), or connectivity that is partly random and irregular despite having statistical structure and regularity (see, *e*.*g*., Ref. [4–10]). However, a general effective methodology for fitting such models to experimental data, such as heterogeneous samples of biological tuning curves is lacking.

A related central problem in neural data analysis is that of inferring functional and synaptic connectivity from neural responses and correlations. A rich literature has addressed this problem [11–16]. However, we see two shortcomings in previous approaches. First, most methods are based on forward models originally developed in statistics that are primarily inspired by their ease of optimization and fitting to data, rather than purely by theoretical or biological principles. Second, in the vast majority of approaches, the outcome is the estimate of the particular connectivity matrix between the particular subset of neurons sampled and simultaneously recorded in a specific animal [11–16]. Post-hoc analyses may then be applied to such estimates to characterize various statistical properties and regularities of connectivity [12, 16]. However, such statistical properties are, in most cases, the object of scientific interest, as they generalize beyond the specific recorded sample. Examples of such statistical properties are the dependence of connection probability between neurons on their physical distance [17] or preferred stimulus features [18]. Another example is the degree to which neuron pairs tend to be connected bidirectionally beyond chance [19]. A methodology for model-based inference of such circuit properties directly from simultaneously or non-simultaneously recorded neural responses is lacking.

Here we propose a methodology that is able to fit theoretically motivated circuit models to recorded neural responses, and infer model parameters that characterize the *statistics* of connectivity or of single-cell properties. Conceptually, we propose to view network models with heterogeneity and random connectivity as generative models for the observed neural data, *e*.*g*., a model that generates diverse tuning curves and hence implicitly models their (high-dimensional) distribution.

The generative model is determined by a set of network parameters which specify the distribution of structural circuit variables like individual synaptic connections or single-cell biophysical properties. In this picture, the particular realization of the connectivity matrix or of biological properties of particular neurons are viewed as latent variables. Traditional, likelihood-based approaches such as expectation-maximization or related approaches need to fit or marginalize out (*e*.*g*., using variational or Monte Carlo sampling methods) such latent variables, conditioned on the particular observed data sample. Such high-dimensional optimizations or integrations are computationally very expensive and often intractable.

Alternatively, one could fit theoretical circuit models by approaches similar to moment matching, or its Bayesian counterpart, Approximate Bayesian Computation [20, 21]. In such approaches, one *a priori* comes up with a few summary statistics, perhaps motivated on theoretical grounds, which characterize the data objects (*e*.*g*., tuning curves). Then one tunes (or in the Bayesian case, samples) the model parameters (but not latent variables) so that the few selected summary statistics are approximately matched between generated tuning curve samples and experimental ones [4]. This approach will, however, generally be biased by the *a priori* choice of the fit summary statistics, and does not exploit all the information available in the data for inferring circuit properties.

A suite of new methods have recently been developed in machine learning for fitting *implicit* generative models [22–24], *i*.*e*., generative models for which a closed or tractable expression for the likelihood or its gradient is not available. Here, we will demonstrate that a specific class of such methods, namely Generative Adversarial Networks (GANs) [23, 25, 26], can address the above problems. In particular, compared to methods such as moment matching, our proposed approach fits the entire high-dimensional data distribution in a much more unbiased and data-driven manner and without the need to choose a few summary statistics *a priori*. As we will show, this results in a more accurate and robust inference of circuit properties. In addition to inferring circuit parameters, this approach also allows for a more unbiased model comparison: one can simply simulate the competing circuit models, after fitting them to training data, and compare their goodness of fit, possibly to unseen data including new stimulus conditions not covered in the tuning curves used for training.

The rest of this article is organized as follows. We start the Results section by introducing the conceptual view of circuit models as implicit generative models for tuning curves. We then introduce the GAN framework. Next, we present the results of applying GANs to fit and infer the parameters of two recent influential circuit models from theoretical neuroscience. In Experiment 1 we fit a feedforward model of motor cortex to an experimental dataset of hand-position tuning curves, and compare the match between the distributions of empirical tuning curves and those generated by the models fit using our proposed method and traditional moment matching. In Experiments 2 and 3 we apply the method to fit a recurrent network model of visual cortex to a simulated dataset of stimulus-size tuning curves generated by a ground-truth circuit model. In Experiment 2 we show that the fit model captures the statistics of observed tuning curves very accurately. In Experiment 3, we assess the accuracy of circuit parameter identification using our proposed method, and discuss factors (such as the kind of tuning curves used for inference) that affect it. In the Discussion we conclude by discussing areas for extension and improvement of our proposed methodology, and broader potential applications of it. The details of our experiments, algorithms, and the models are given in Materials and methods; the source code for all implemented examples is available from https://github.com/ahmadianlab/tc-gan under the MIT license.

## Results

### Mechanistic network models as implicit generative models

We consider mechanistic network models of the type developed in theoretical neuroscience, informed by knowledge of biological mechanisms and network anatomy, or by computational principles. We limit ourselves to networks evolving according to feedforward or recurrent firing rate equations (examples include Ref. [2, 4–10, 27, 28]), although the methodology is extendable to spiking networks as well (possibly with slight modifications to ensure differentiability). In the general presentation of this subsection we focus on recurrent rate networks. Abstractly, the neural responses in such networks evolve according to a dynamical system that has the following general structure (for a concrete example see the model in Experiment 2 below, which is governed by Eq (5)):

$$\begin{array}{c} \frac{dv_{t}}{dt}=F\left(v_{t}\;;\;I,W,\gamma \right). \end{array}$$ (1)

Here, **v**_*t*_ is the vector of the state variables of the network’s neurons at time *t* (components of **v**_*t*_ may, *e*.*g*., include the firing rate, membrane voltage and other “fast” state variables of individual neurons or synapses) and **F** is a vector field on state space which is differentiable with respect to its arguments. The matrix **W** is the partially disordered synaptic connectivity matrix (which can include both the recurrent, as well as feedforward external connections), and **γ** is the vector of possibly heterogeneous single-cell biophysical constants (*e*.*g*., membrane time-constant, spiking threshold potential, parameters of input-output nonlinearity, etc.). Finally, the vector **I** is the external input to the network which can represent stimuli or state-dependent modulators; we let **I** depend on a discrete index variable *s* denoting the stimulus or experimental condition (or discretized parameter). **I**(*s*) can in general be time-dependent, but here we assume it is stationary for simplicity. We also assume that **I**(*s*) is deterministic, and that all quenched randomness in network structure is captured by **W** and **γ**.

**Fig 1:**
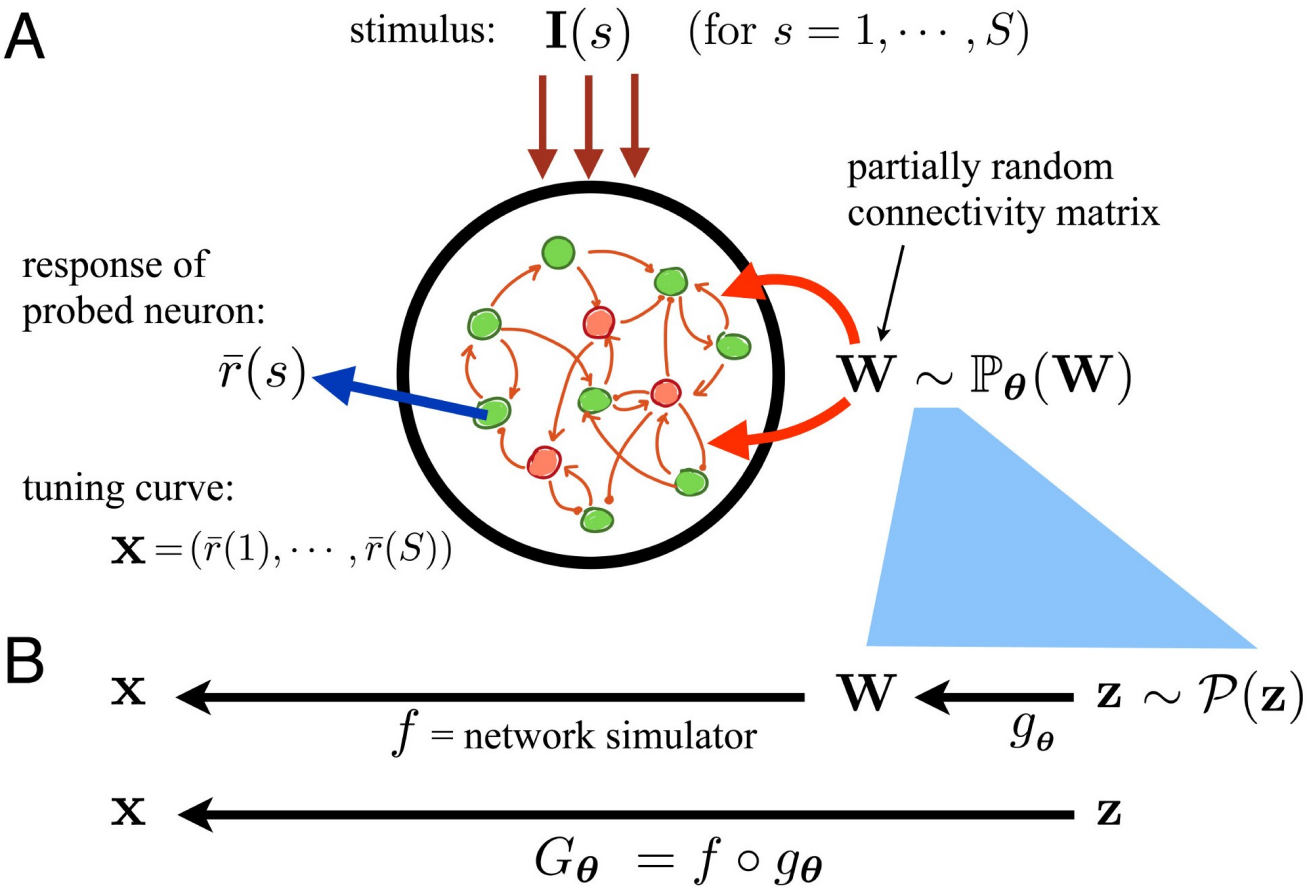
Viewing mechanistic neuronal circuit models as generative models for tuning curves. (A) Schematic representation of a generic recurrent circuit model from theoretical neuroscience. The network structure, in this case reduced to the full recurrent connectivity matrix **W**, is partially random with statistics described by the distribution $P_{\theta }\left(W\right)$, which depends on parameters ***θ***. These parameters could, *e*.*g*., include the average strength or spatial range of connections between different cell types. The network receives an external input, **I**(*s*), that represents stimuli, which can be in one of *S* different conditions employed in an experiment. The output of the model is taken to be the sustained response, $\bar{r}\left(s\right)$, of a pre-selected probe neuron (blue arrow). Given a realization of the network structure **W** (sampled from $P_{\theta }\left(W\right)$), this response can be obtained in each stimulus condition, *s*, by simulating the network in the presence of external input **I**(*s*). The responses, $\bar{r}\left(s\right)$, for all stimulus conditions are then concatenated into a tuning curve vector $x=(\bar{r}\left(1\right),\cdots ,\bar{r}\left(s\right))$, which is the ultimate output of the network when viewed as a generative model. (B) The deterministic mapping *f* stands for the simulation process that links the particular realization of the network structure **W** to its functional (tuning curve) output **x**. Since the ultimate goal is to use gradient-based methods to learn the model parameters, ***θ***, the process of sampling a realization of **W** is cast (cyan area) using a parametrized mapping, *g*_***θ***_, that transforms a set of standard noise variables **z**, sampled from a fixed distribution, into **W**. The composition of *f* and *g*_***θ***_ yields the full parametrized generator function, *G*_***θ***_ ≡ *f* ◦ *g*_***θ***_, as used in the GAN framework (bottom row). Given a set of parameters, the generator thus receives a set of noise variables, **z**, sampled from their standard distribution, and generates a tuning curve, **x**.

In order to exploit tuning curve datasets to constrain mechanistic network models, and in the process make model-based inferences about circuit properties, we propose to view network models of the above type as generative models for tuning curves. This view, which we will expound below, is graphically summarized in Fig 1. (Even though here we take trial-averaged tuning curves as the ultimate functional output of the model, this is not central to our proposed view; as discussed at the end of the Discussion, other quantities characterizing neural activity such as pairwise correlations or higher-order population statistics can augment or replace tuning curves in general applications.)

Given a choice of the non-dynamical variables **W**, **γ** and **I**(*s*), and the initial condition **v**(*t* = 0) (*t* = 0 can, *e*.*g*., be the stimulus onset time), the network dynamics can be simulated to compute the full temporal trajectory of all neural state variables. From this simulated trajectory, the average response, $\bar{r}\left(s\right)$, of a designated “probe” neuron in the network during a given “response interval” can be calculated in each condition *s* (this is the time-average of a certain component of **v**_*t*_ for *t* in the response interval). The vector $x\equiv (\bar{r}\left(1\right),\cdots ,\bar{r}\left(S\right))$ is then the tuning curve of that neuron, containing its responses in *S* different stimulus conditions which we assume are present in the training data. (Note that once a network model is trained, it can be applied to stimuli other than those used for training.) Thus, for networks with deterministic dynamics considered here, there is a deterministic mapping between the tuple of network’s structural variables, (**W**, **γ**), and the tuning curve of a given network neuron (we assume fixed initial conditions, **v**_*t*=0_, or otherwise ignore the dependence of output on them). We call this mapping *f* (see Fig 1).

Note that **γ** and **W** typically have very large dimensions; for a network of *N* neurons, **γ** and **W** have on the order of *N* and *N*^2^ components, respectively. In the proposed methodology, these large sets of structural and physiological network constants should be viewed as latent variables rather than model *parameters* that are fit to data. We are interested in cases in which these heterogeneous high-dimensional vectors are sampled from statistical ensembles (distributions) that capture the structured regularities as well as disordered randomness in single-cell properties and network connectivity. Consider a statistical ensemble described by a parameterized distribution $P_{\theta }\left(W,\gamma \right)$. Through the deterministic map, *f*, between (**W**, **γ**) and **x**, this distribution in turn induces a distribution, $P_{\theta }\left(x\right)$, over the tuning curve **x**, which is also parameterized by ***θ***. The model’s parameter vector, ***θ***, which is typically low-dimensional (see the examples in Experiment 1–3 subsections below), determines the network’s statistical structure and constitutes the parameters that we would like to fit to data. Components of ***θ*** can control, *e*.*g*., the average strength or spatial range of synaptic connections, or the mean and dispersion of biophysical single-cell properties.

Traditional likelihood-based methods infer the circuit properties, encapsulated in ***θ***, by maximizing the likelihood function $P_{\theta }\left({\left(x_{i}\right)}_{i=1}^{N}\right)\approx \prod_{i}P_{\theta }\left(x_{i}\right)$ given a dataset of tuning curves ${\left(x_{i}\right)}_{i=1}^{N}$. However, for cases of interest, the mapping *f* is typically very complex and practically cannot be inverted (even though it can be relatively cheaply simulated in the forward direction); therefore in practice $P_{\theta }\left(x\right)$ cannot be computed explicitly. Moreover, most likelihood-based methods are based on expectation-maximization-like algorithms; in the expectation step, such algorithms have to infer the high-dimensional latent variables (**W**, **γ**), which is a highly expensive computation.

In the next subsection we discuss how recently developed methods in machine learning, in particular generative adversarial networks, can be used to fit generative models of the above type, for which the parametrized data distribution, $P_{\theta }\left(x\right)$, is only implicitly defined. All that is required in those approaches is the generative process that, given a random seed, generates a tuning curve (or a set of tuning curves), via a function that is differentiable with respect to ***θ***. More formally, in such frameworks the generative model, or the “generator”, is characterized by a parametrized function *G*_***θ***_ which, given an input vector, **z**, of random noise variables that have a *fixed* or standard distribution, outputs a tuning curve **x** = *G*_***θ***_ (**z**). This is almost identical to the case of mechanistic circuit models described above, with two technical differences. First, the generative network process, as described above and in Fig 1, is captured by the function *f* which does not directly depend on the model parameters, ***θ***. Instead, it is the inputs to this function, namely (**W**, **γ**), which implicitly depend on ***θ***, as they are sampled from $P_{\theta }\left(w,\gamma \right)$. Thus the second difference is that the inputs to *f*, *i*.*e*., the network’s structural variables (**W**, **γ**), do not have a fixed standard distribution, but rather a distribution dependent on ***θ***.

However, we can use the so-called “reparametrization trick” [22], to remedy this mismatch and cast the circuit-model-based generative processes of interest to us in the required form. To this end, we will formulate the sampling of (**W**, **γ**) from their statistical ensemble via a deterministic function or mapping, *g*_***θ***_, parametrized by ***θ***, that receives the fixed-distribution noise variables **z** as input. For example, a synpatic weight, *w*, with a gaussian distribution parametrized by its mean, *μ*, and variance, *σ*^2^, can be generated by the function *g*_***θ***_ (*z*) ≡ *μ* + *σz* where *z* is sampled from the standard (zero-mean, unit-variance) normal distribution, and ***θ*** = (*μ*, *σ*) in this case. We provide biologically relevant examples of *g*_***θ***_ in Materials and methods (see Eqs (17)–(19), (24) and (26)). Note that in the typical application of interest to us, while ***θ*** is low-dimensional, **z** has high dimensionality, on the order of the dimensions of (**W**, **γ**). The full generator function *G*_***θ***_ is then simply the composition of *f* and *g*_***θ***_: *G*_***θ***_ (**z**) ≡ *f* (*g*_***θ***_ (**z**)) (see Fig 1B). In other words, first the standard noise variables **z** and network parameters ***θ*** together determine the full particular realization of network structure. The network is then simulated and a tuning curve (or a set of tuning curves) is generated. The function *f* is typically differentiable, and for many statistical ensembles of interest the function *g*_***θ***_ is (or can be closely approximated by a) function that is differentiable with respect to ***θ***. Then *G*_***θ***_ will also be differentiable in ***θ***. As we describe in the rest of the article, with this formulation, we can use methods like generative adversarial networks to fit mechanistic neuronal circuit models to datasets of tuning curves.

### Generative Adversarial Networks

Generative Adversarial Networks (GANs) are a framework for training generative models developed by the deep learning community [23, 29, 30]. The GAN approach is powerful because it is applicable to implicit generative models, *e*.*g*., the mechanistic networks discussed in the previous subsection, for which evaluating the likelihood function or its gradient is intractable. Another advantage of GANs (in the context of the previous subsection) is that, unlike typical likelihood-based methods, they fit the model parameters ***θ*** directly, skipping the computationally costly step of inferring the high-dimensional latent variables, namely the particular realization of network connectivity matrix **W** or single-cell constants **γ**. Note that unlike the particular realization of the connectivity matrix between the experimentally sampled cells, the model parameters ***θ***, which characterize the *statistics* of connectivity and single-cell properties, are generalizable and of direct scientific interest.

All that is required in the GAN approach is a generative process that, given a random seed, generates a sample data object, via a function that is differentiable with respect to ***θ***. While in machine learning applications the generated data object is often an image (and the generative model is a model of natural images), in our case it will be a tuning curve, formalized in the Introduction as the vector $x\in R^{S}$ containing the trial-averaged responses of a probed network neuron in *S* different experimental conditions (*e*.*g*., *S* different values of a stimulus parameter).

In a GAN there are two networks: a “generator” and a “discriminator”. The generator implements the generative model and generates sample data objects, while the discriminator (which can be a classifier) distinguishes true empirical data objects from “fake” ones generated by the generative model. Conceptually, the discriminator and generator compete: the discriminator is trained to better distinguish real from fake data, and the generator is trained to fool the discriminator.

More formally, the generator is characterized by a parametrized function *G*_***θ***_ which, given an input vector of random noise variables **z** that have a *fixed* distribution, outputs a sample data object **x** = *G*_***θ***_ (**z**). As we saw in the previous subsection, the process of generating a tuning curve by mechanistic neuronal circuit models can also be formulated in this manner (see Fig 1). In that case, the vector **z** provides the random seed for the generation of the full network structure (*i*.*e*., all synaptic weights and single-cell biophysical constants), given which the network is simulated to generated an output tuning curve **x**. We note that while in our applications the generator parameters ***θ*** usually correspond to physiological and anatomical parameters with clear mechanistic interpretations, in typical machine learning usage the structure of the GAN generator (*e*.*g*., a deconvolutional deep feedforward network, generating natural images) and its parameters may have no direct mechanistic interpretation (see Materials and methods, under “Differences with common machine learning applications”, for further discussion of this point).

The second network in a GAN is the discriminator. Mathematically, the discriminator is a function $D_{w}$, parametrized by ***w***, that receives a data object (in our case, a tuning curve) as input, and outputs a scalar. $D_{w}$ is trained so that its output maximally discriminates between the real data samples and those generated by *G*_***θ***_. The generator is in turn trained to fool the discriminator. If the discriminator network is sufficiently flexible, the only way for the generator to fool it is to generate samples that effectively have the same joint distribution as real data. When $D_{w}$ is differentiable in its input and parameters and *G*_***θ***_ is differentiable in its parameters, training can be done using gradient-based algorithms. GANs can nevertheless be difficult to train and many techniques have been developed to improve their training. In this work we employ the Wasserstein GAN (WGAN) approach which has shown promise in overcoming some of the shortcomings of the traditional GAN approach [25, 26]. (We note, however, that traditional GANs could also be used for the types of application we have in mind.) The WGAN approach is mathematically motivated by minimizing the Wasserstein or earth mover’s distance between the empirical data distribution and the distribution of the generator output [25]. The Wasserstein distance provides a measure of similarity between distributions which (unlike *e*.*g*., the Kullback-Leibler divergence, which is the distance minimized in maximum-likelihood fitting) exploits the metric or similarity structure in the data space $R^{S}$.

In the context of WGANs, the discriminator can be viewed as yielding a *scalar* measure or “summary statistic” for a (typically high-dimensional) data object or tuning curve. A single, fixed summary statistic D(x) can be used to measure the divergence between two distributions as the difference between the expectation of D(x) under the two distributions. However, two distributions can lead to the same average D(x) and yet be completely different in other respects. For example, consider the case of orientation tuning curves for visual cortical neurons. An example D is the function that receives an orientation tuning curve as input and outputs the half-width of that tuning curve (which measures the strength of orientation tuning). A generative model may produce orientation tuning curves that on average are narrower (or broader) than the average empirical tuning curve. However, even when a model matches the data distribution of tuning curve widths, its generated tuning curves may look very different from true ones along other dimensions (*e*.*g*., along the average height or maximum response dimension, or in the variance of tuning widths). One interpretation of the WGAN methodology is that instead of looking at data objects (tuning curves) along a fixed dimension using a fixed scalar measure, it optimizes that measure or probe to maximally distinguish between model-generated *vs*. empirical data objects. (In other flavors of GAN, the discriminator has other useful interpretations and can provide an estimate of the density of generator output in data space relative to the true data distribution; see Materials and methods, under “Alternatives to WGAN, and alternative views of GANs”.)

Let S be the class of all possible “smooth summary statistics” that can be used to characterize tuning curves; more technically, S is taken to be the set of all *scalar* functions of tuning curves, $D:R^{S}\to R$, that are Lipshitz-continuous with a Lipshitz constant less than one (if D is differentiable, the latter condition is equivalent to constraining the gradient of D to have norm less than one everywhere). Interestingly, by the Kantorovich-Rubinstein duality [25, 31], the earth mover’s distance, *d*(*ρ*, *ν*), between two distributions, *ρ*(**x**) and *ν*(**x**), can be expressed as the difference between the expectations of a *maximally discriminating* smooth summary statistic under the two distributions:

$$\begin{array}{c} d\left(\rho ,\nu \right)=\max_{D\in S}|E_{x∼\rho }\left[D\left(x\right)\right]-E_{x∼\nu }\left[D\left(x\right)\right]|. \end{array}$$ (2)

In our applications, one distribution (say *ν*) is the data distribution, and the other (say *ρ*) is the output distribution of *G*_***θ***_ which we would like to move closer to the data distribution. This can thus be done by iterating between improving D to maximize $\left|E_{x∼\rho }\left[D\left(x\right)\right]-E_{x∼\nu }\left[D\left(x\right)\right]\right|$, and improving *G*_***θ***_ to minimize it.

To obtain a practical algorithm that in this manner approximately minimizes the earth mover’s distance, Eq (2), the expectations over the two distributions are approximated by mini-batch sample averages, and the discriminator class S is approximated by single-output feedforward neural network (parametrized by weight vector ***w***) with input-output gradients of norm less than one (such networks form a proper subset of S; in practice, the gradient norm is only forced to be close to one). This gives rise to an adversarial algorithm in which the discriminator $D_{w}$ and the generator *G*_***θ***_ are trained by iteratively alternating between minimizing the following two loss functions

$${\text{Loss}}_{D}\left(w,\theta \right)=E_{z}\left[D_{w}\left(G_{\theta }\left(z\right)\right)\right]-E_{x}\left[D_{w}\left(x\right)\right]+\;\left(\text{Gradient}\;\text{Penalty}\right)$$ (3)

$${\text{Loss}}_{G}\left(w,\theta \right)=-E_{z}\left[D_{w}\left(G_{\theta }\left(z\right)\right)\right],$$ (4)

where Eq 3 is minimized with respect to the discriminator’s parameters (***w***), and Eq 4 is minimized with respect to the generator’s parameters (***θ***). Here $E_{x}$ and $E_{z}$ denote averages over a batch of empirical data samples and samples from the standard noise distribution, respectively (thus $E_{z}\left[D_{w}\left(G_{\theta }\left(z\right)\right)\right]$ is the same as the average of $D_{w}\left(x\right)$ when **x** is sampled from the generator output, instead of the empirical data distribution). The “Gradient Penalty” term forces the gradient of $D_{w}$ to be close to one (see Materials and methods, under “Conditional Generative Adversarial Networks”, for details). Finally, a penalty term Penalty_*G*_(***θ***) can be added to the generator loss, Eq (4), as a regularization term for generator parameters.

#### Connection with moment matching

Here we point out a connection between the WGAN and the moment matching approach to model fitting. In the simplest version of moment matching, a certain scalar statistic of interest, M(x), that evaluates on data objects, is first chosen. The model parameters are then fit by matching the expectations or averages of that statistic (the “moments”) under the empirical and generative model distributions. This can be done *e*.*g*., by minimizing $d_{M}\left(\rho ,\nu \right)\equiv |E_{x∼\rho }\left[M\left(x\right)\right]-E_{x∼\nu }\left[M\left(x\right)\right]|$ (in practice the squared difference is usually minimized). Comparing this equation with the Wasserstein distance Eq (2) underlying WGAN, we see that (at least in the case of matching a single moment) moment matching is equivalent to using a pre-defined and fixed discriminator D=M. By contrast, in the GAN approach the discriminator is not pre-determined, but is optimally chosen from a large and flexible class of discriminator functions to maximally distinguish true *vs*. generated data objects. An intermediate method for fitting implicit generative models is provided by Generative Moment Matching Networks [32] which can be thought of as a kernel-based generalization of moment matching.

#### Conditional GANs

In the original GAN formulation, the discriminator only receives the generator’s output, which in our case is a tuning curve, $x=(\bar{r}\left(1\right),\cdots ,\bar{r}\left(S\right))$, defined as the joint responses of a neuron across *S* different stimulus conditions. In particular, this means that all tuning curves in the training dataset must contain responses in all of the same *S* conditions, as the discriminator input size cannot vary. Any empirical tuning curve with missing conditions must be discarded from the experimental dataset.

These shortcomings are ameliorated by using a so-called conditional GAN [33]. In our setting, the conditional GAN’s discriminator receives as input not only the neuronal responses, **x**, but also a subset of the experimental or stimulus parameters at which those responses were recorded. Since the discriminator only sees the responses at one value of the explicitly provided experimental parameters at a time, not every neuron has to be recorded for all choices of those parameters. For example, in Experiment 3 below, we consider the case of primary visual cortical neurons whose responses to grating stimuli of different sizes are recorded, but the grating center is allowed to have a nonzero offset from the neuron’s receptive field center. If we consider the offset as the explicit condition for the conditional GAN, we can include size tuning curves in the dataset that contain recorded responses at all stimulus sizes, but not at all possible offsets.

In what follows we use both the original WGAN formulation (in Experiments 1–3), and later the conditional WGAN (cWGAN; in Experiment 3) to demonstrate the power of the latter method to fit a simulated dataset with diverse stimulus conditions.

### Experiment 1: Complex M1 tuning curves

In this subsection and the next two, we illustrate the GAN-based model fitting approach by applying it to two previously published mechanistic circuit models developed in theoretical neuroscience to explain various nonlinear features of cortical responses.

As our first example we take a feedforward model of primary motor cortex (M1) tuning curves proposed by Ref. [4]. The tuning curves describe the response tuning of M1 neurons as a function of 3D hand position and posture (supination or pronation). The model was proposed as a simple plausible mechanism for generating the significant complex nonlinear deviations of observed monkey M1 tuning curves from the classical linear model [34].

**Fig 2:**
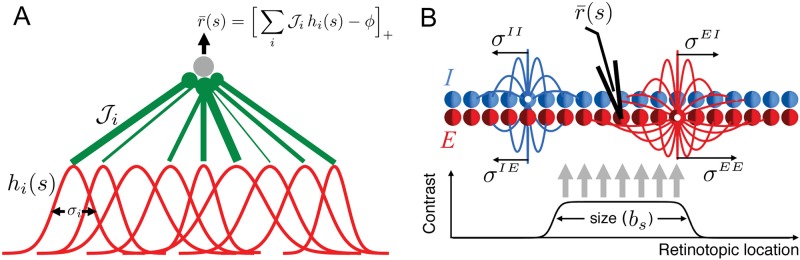
Structure of the feedforward and recurrent generative models used in our computational experiments. (A) The feedforward network model of primary motor cortex (M1) is borrowed from Ref. [4] and produces heterogeneous hand-location tuning curves. This heterogeneity is rooted in the random network structure, including the variability in the input layer (modeling a premotor or parietal area) receptive field widths, *σ*_*i*_, feedforward weights to the M1 layer, $J_{i}$, and the threshold, *ϕ*, of M1 output neurons which are rectified linear units. (B) The structure of the Stabilized Supralinear Network (SSN) with one-dimensional topography (retinotopy) as a model of the primary visual cortex (V1). The SSN is a recurrent network of excitatory (*E*) and inhibitory (*I*) neurons. The visual stimulus (bottom) models the input to V1 due to a grating of diameter *b*_*s*_ in condition *s*. Heterogeneity in model output (size tuning curves) originates in the heterogeneity of feedforward and recurrent horizontal connections. The mean and variance of the horizontal connections between SSN neurons depend on the pre- and postsynaptic cell-types and their retinotopic distance, and for different connection-types falloff over different characteristic length scales. For a full description of models and their parameters see Materials and methods.

We used the “extended” model of Ref. [4] with small modifications (see Materials and methods subsection “Feedforward Model of M1”). In particular, we did not model response tuning with respect to hand posture (we did this in order to increase the size of the dataset by combining hand-position tuning curves from both posture conditions to allow for proper cross-validated testing of model fits). The model is a two-layer feedforward network, with the input layer putatively corresponding to the parietal reach area or to premotor cortex, and the output layer modeling M1 (see Fig 2). The input layer neurons’ activations are given by 3D Gaussian tuning curves defined on the hand position space. The receptive field centers formed a fine regular grid, but their widths varied randomly across the input layer, and were sampled independently from the uniform distribution on the range [*σ*_*l*_, *σ*_*l*_ + *δσ*] (see Fig 2A). The feedforward connections from the input to output layer are random and sparse, with a connection probability of 0.01. In our implementation of this model, the strength of the nonzero connections were sampled independently from the uniform distribution on the range [0, *J*]. The response of an output layer neuron is given by a rectified linear response function with a threshold. The thresholds were allowed to be heterogeneous across the M1 layer, and were sampled independently from the uniform distribution on the range [*ϕ*_*l*_, *ϕ*_*l*_ + *δϕ*]. Thus in total the model has five trainable parameters ***θ*** = (*σ*_*l*_, *δσ*, *J*, *ϕ*_*l*_, *δϕ*).

Using the WGAN framework, we fit the five model parameters to a dataset of experimentally measured monkey M1 tuning curves recorded by Ref. [4] and available online. With hand-posture conditions ignored, the tuning curves in this dataset describe the trial-averaged responses of a given M1 neuron in 27 experimental conditions corresponding to the monkey holding its hand in one location out of a 3 × 3 × 3 cubic grid of 3D spatial locations. (We ignored the hand-position label by blindly mixing hand-position tuning curves across pronation and supination conditions, as if they belonged to different neurons.) We randomly selected half of the hand-position tuning curves (*n* = 257) to be used for training the model, and used the other half (*n* = 258) as held-out data to evaluate the goodness of model fits.

**Fig 3:**
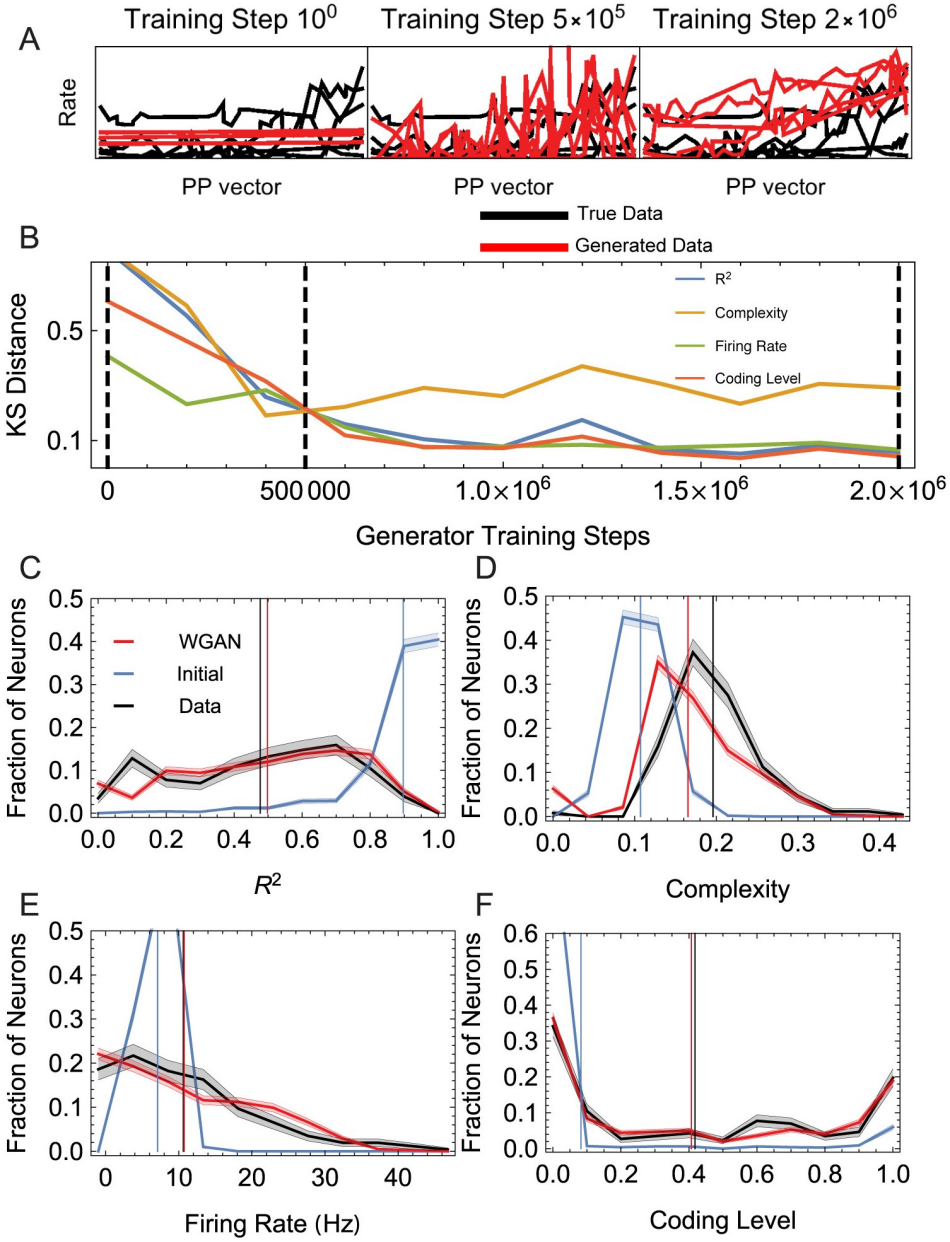
Summary of the feedforward model fit to the M1 tuning curve dataset of Ref. [4]. (A) Data and model tuning curves at different stages of training; the plotted tuning curves are the projections of the 3D tuning curves along the preferred position (PP) vector which is obtained by a linear fit to the 3D tuning curve (see Ref. [4]). (B) Kolmogorov-Smirnov (KS) distance throughout training between the data and model distributions, shown in panels C–F, of four summary statistics characterizing the tuning curves evaluated on held-out data. Vertical dashed lines mark the epochs at which curves in panel A are sampled. (C–F) histograms of four tuning curve statistics (*R*^2^, complexity score, firing rates, and coding level) showing the data distribution (black), the initial model distribution (blue) and the final model distribution after fitting (red). Vertical lines show the mean value of each histogram.

The results showing the performance of the fit model are summarized in Fig 3. The figure shows the data and trained model histograms for four test statistics or measures characterizing the tuning curves or responses: firing rate (across the 27 conditions), coding level, *i*.*e*., the fraction of conditions with rate significantly greater than zero (which we took to mean larger than 5 Hz), the *R*^2^ or coefficient of determination of the optimal linear fit to the tuning curve, and the complexity score. Ref. [4] defined the complexity score of a tuning curve to be the standard deviation of the absolute response differences between nearest neighbor locations on the 3 × 3 × 3 lattice of hand positions (the tuning curve was first normalized so that its responses ranged from −1 to 1).

The last two statistics, the *R*^2^ and the complexity score, are of particular theoretical interest, as they provide two measures for the degree of irregular nonlinearity in the tuning curve and thus deviation from the classical linear model of M1 tuning curves [34]. Therefore, Ref. [4] fit their model by matching the mean and standard deviation of these two statistics between model and M1 data. By contrast, as described in the previous subsection, the WGAN, employed here, optimizes the discriminatory statistic, D, and (for complex enough discriminator networks) seeks to fit the entire joint multi-dimensional (in this case 27-dimensional) tuning curve distribution.

We measured the mismatch between the model and data distributions for each of the four test statistics using the Kolmogorov-Smirnov (KS) distance. As shown in Fig 3B, the goodness of fit improves fast during training. The goodness-of-the-fit of the distributions of complexity score and *R*^2^ (Fig 3C and 3D) are comparable to those obtained in Ref. [4] by grid search. It is also notable that the trained model fits the distribution of firing rates and coding levels (Fig 3E and 3F) quite well. In Ref. [4], the authors chose to individually normalize the model and data tuning curves so that their shape, but not their overall firing rate scale, was fit to data. We did not normalize the tuning curves, but as described above added a tunable parameter, *J*, to the model that scales the feedforward connection strengths. We found that with this addition and without normalization, the model is actually capable of accounting for the variability of firing rates across neurons and conditions as well. In the original model of Ref. [4] the thresholds were chosen separately for each output layer neuron such that the coding level of its response matched that of a randomly selected recorded M1 neuron. By contrast, in order to have all structural variability in the model in a differentiable form, we did not fit individual thresholds, but allowed them to be randomly distributed and only fit the two parameters of their distribution, *ϕ*_*l*_ and *δϕ*_*l*_. Even though we did not match coding levels neuron-by-neuron by adjusting individual neural thresholds, the model was able to match the distribution of neural coding levels in the dataset, without the need for tuning individual thresholds.

### Experiment 2: Stabilized Supralinear Network

The second network model we consider is a recurrent model of local cortical circuitry, the Stabilized Supralinear Network (SSN), which has found broad success in mechanistically explaining a range of nonlinear modulations of neural responses and variability by sensory context or attention, in multiple cortical areas [8, 35, 36]. The SSN is a recurrent rate network of excitatory (*E*) and inhibitory (*I*) neurons which have a supralinear rectified power-law input-output function, $f\left(u\right)=k{\left[u\right]}_{+}^{n}$ (where *u* is the total input to a cell, *k* > 0 and *n* > 1 are constants, and [*u*]_+_ = max(0, *u*) denotes rectification). The dynamical state of the network of *N* neurons is the vector of firing rates $r\in R^{N}$ which is governed by the differential equation

$$\begin{array}{c} \tau \frac{dr}{dt}=-r+k[Wr+FI(s){]}_{+}^{n}, \end{array}$$ (5)

where *W* and *F* denote the recurrent and feedforward weight matrices (with structure described below), the diagonal matrix $\tau =\text{Diag}\;({\left(\tau_{i}\right)}_{i=1}^{N})$ contains the neural relaxation time constants, *τ*_*i*_, and **I**(*s*) denotes the stimulus input in condition *s* ∈ {1, …, *S*}.

We consider a topographically organized version of SSN with a one-dimensional topographic map which could correspond, *e*.*g*., to a one-dimensional reduction of the retinotopic map in primary visual cortex (V1). We adopt this interpretation here, and simulate the fitting of an SSN model of V1 to a dataset of V1 grating-size tuning curves, which are commonly used to characterize surround suppression [8, 37, 38]. The model has a neuron of each type, *E* and *I*, at each topographic spatial location. For the *i*-th model neuron, we denote its type by *α*(*i*) ∈ {*E*, *I*} and its topographic location by *x*_*i*_ (which ranged from −0.5 to 0.5 on a regular grid).

In many cortical areas the statistics of local recurrent connectivity, such as connection probability and average strength, systematically depend on several factors, including the types of the pre- and post-synaptic neurons, the physical distance between them, or the difference between their preferred stimulus features [17, 18]. We made a choice for the distribution of *W*_*ij*_ (the connection strength from neuron *j* to neuron *i*) that accounts for such dependencies in our topographic network, and also respects Dale’s principle [39, 40]. In order to make the GAN method applicable, another criteria was that *W*_*ij*_ are differentiable with respect to the parameters of their distribution. The most relevant aspects of the distribution of *W*_*ij*_ for the network dynamics are its first two moments (equivalently, mean and variance). We assumed that *W*_*ij*_’s are independent with uniform distributions on the range [〈*W*_*ij*_〉 − *δW*_*ij*_/2, 〈*W*_*ij*_〉 + *δW*_*ij*_/2], with a mean, 〈*W*_*ij*_〉, and width, *δW*_*ij*_, that depend on the pre- and post-synaptic types and fall off with the distance between the pre- and post-synaptic neurons over characteristic length scales. More precisely we chose the fall off to be Gaussian, and set

$$\left\langle W_{ij}\right\rangle =\pm {\bar{J}}_{ab}\;\text{exp}\;(-\frac{{\left(x_{i}-x_{j}\right)}^{2}}{2\sigma_{ab}^{2}})$$ (6)

$$\delta W_{ij}=\delta J_{ab}\;\text{exp}\;(-\frac{{\left(x_{i}-x_{j}\right)}^{2}}{2\sigma_{ab}^{2}})$$ (7)

where *a* = *α*(*i*) and *b* = *α*(*j*) are the *E*/*I* types of the neurons *i* and *j*, respectively. The 2 × 2 matrices ${\bar{J}}_{ab}$, *δJ*_*ab*_, and *σ*_*ab*_ constitute 12 trainable model parameters; they control the average strength, the degree of random heterogeneity, and the spatial range of the recurrent horizontal connections between different *E*/*I* cell types, respectively. All parameters are constrained to be non-negative, and we also enforced the constraint ${\bar{J}}_{ab}\geq \delta J_{ab}/2$; the sign on the right side of Eq (6), which is positive or negative, when the presynaptic cell type *b* is *E* or *I*, respectively, enforces the correct sign for *W*_*ij*_ according to Dale’s principle.

The feedforward input, *F***I**(*s*), to the SSN is composed of the stimulus-independent feedforward connectivity *F* and the topographically structured visual stimulus **I**(*s*). We chose the matrix *F* to be square and diagonal and hence **I**(*s*) to be *N*-dimensional. To model size tuning curves, we let the visual input **I**(*s*) in condition *s* only target neurons in a central band of the topographic grid roughly extending from location −*b*_*s*_/2 to *b*_*s*_/2 (see Fig 2B), as a simple model of visual input from a grating with diameter *b*_*s*_. We included quenched random heterogeneity in the diagonal feedforward weights by sampling *F*_*ii*_ independently from the uniform two-point distribution on [1 − *V*, 1 + *V*]; thus *V* controls the degree of disorder in the feedforward inputs to network cells.

We modeled size tuning curves based on the sustained response (defined as the time averaged firing rate during the “sustained response” period; see Materials and methods) of a subset of SSN neurons driven by stimuli of different diameters or sizes. In experiments, typically the grating stimulus used to measure a neuron’s size tuning curve is centered on the neuron’s receptive field. To model this stimulus centering, we let the model output, $\bar{r}\left(s\right)$, in condition *s* be the sustained response of the excitatory neuron at the center of the topographic grid (which is also the center of the stimulus with size *b*_*s*_). Note that in general, the tuning curves in random SSN’s show variability across neurons as well as across different realizations of the network for a fixed output neuron. Furthermore, when *N* is large, the variability across the tuning curves of neurons with topographic locations sufficiently near the center often approximates variability of the center neuron across different network realizations with different **z**. Although we do not do so here, this self-averaging property may in principle be exploited for more efficient training of the model.

We used the GAN framework to fit the 12 recurrent connectivity parameters and one feedforward parameter of the SSN model, $\theta =({\bar{J}}_{ab},\delta J_{ab},\sigma_{ab},V)$, to simulated data. The latter consisted of 2048 size tuning curves generated from a “ground truth” SSN with the connectivity parameters listed in Table 1. (We used a slight reparametrization of ***θ*** in the actual WGAN implementation; see Eq (28).) All other model parameters were the same between the ground truth and trained SSN models (see Materials and methods for details).

**Table 1:** The parameter values for the ground-truth SSN used for generating the training set tuning curves. The columns correspond to different (*a*, *b*) possibilities. The feedforward weight heterogeneity parameter is *V* = 0.1.

Parameter	*EE*	*EI*	*IE*	*II*
*J*_*ab*_	0.3	0.5	0.4	0.2
*δJ*_*ab*_	0.3	0.4	0.6	0.19
*σ*_*ab*_	0.15	0.025	0.1	0.025

**Fig 4:**
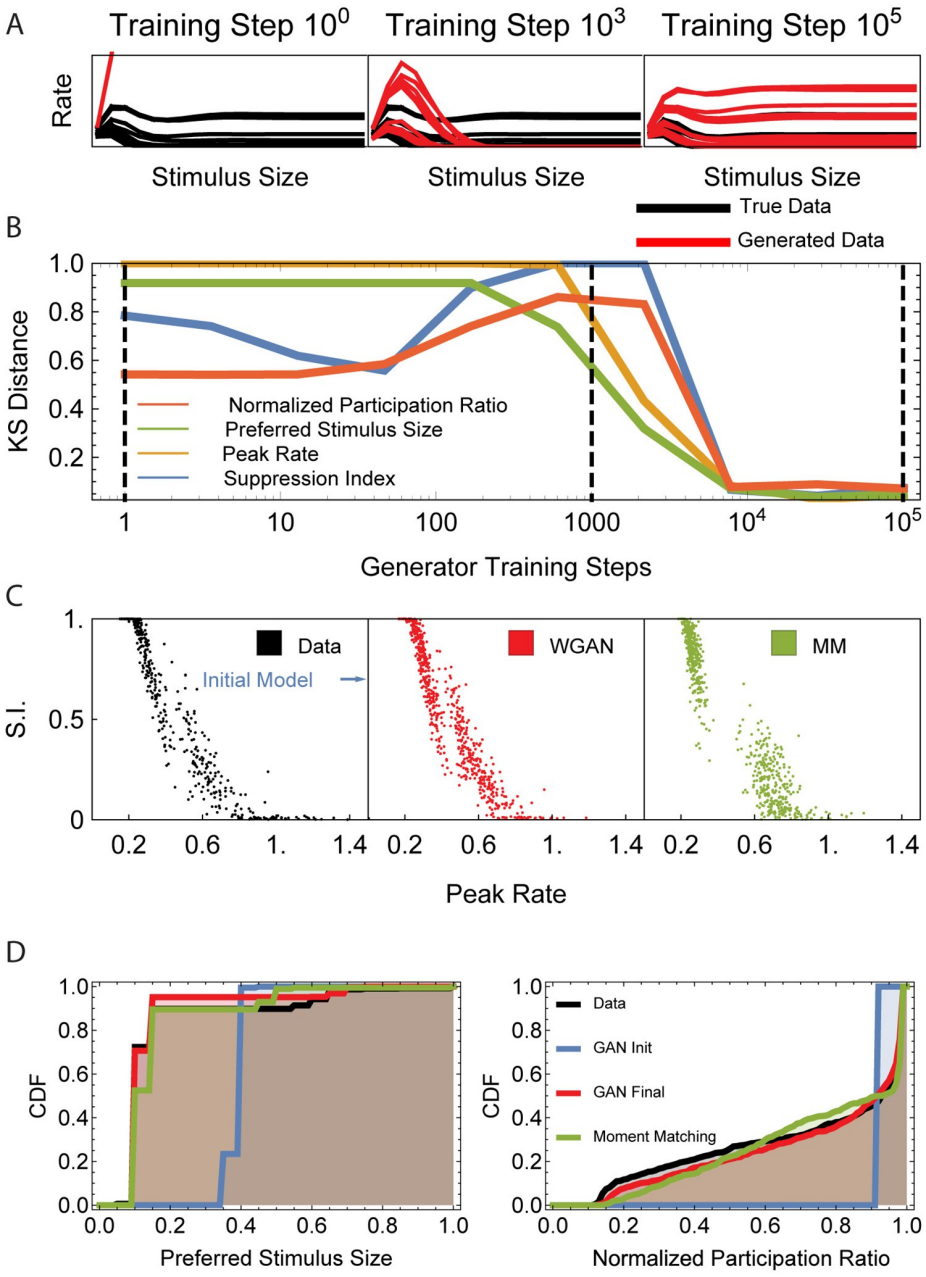
Summary of the recurrent SSN model fit to simulated V1 tuning curve data. (A) Data and model tuning curves at three different stages of training. (B) KS distance between the data and model distributions of four summary statistics characterizing the tuning curves, throughout training. Vertical dashed lines mark the epochs at which curves in panel A are sampled. (C) Scatter plots of peak rate *vs*. suppression index (S.I.) for the training data, the WGAN fit, and moment matching fit. (D) Cumulative distribution functions (CDF) for preferred stimulus size and normalized participation ratio of the data, the WGAN fit, and moment matching.

To quantify the goodness of fit between the data and trained model distributions of tuning curves, as in the previous section, we compare the distributions of four test statistics or measures characterizing the size tuning curves: preferred stimulus size, maximum firing rate, the suppression index, and normalized participation ratio. The preferred stimulus size is the stimulus size eliciting the largest response or maximum firing rate. The suppression index for a neuron (or tuning curve) measures how much the response of that neuron is suppressed at large stimulus sizes relative to response to its preferred size. Finally, the normalized participation ratio measures the fraction of tested stimulus sizes that elicit a response close to the maximum response. (See Materials and methods for the precise definition). Note that while to fit the model we used size tuning curves containing responses to stimuli with *S* = 8 different sizes, for testing purposes, we generated tuning curves from the trained and ground-truth SSN’s using a larger set of stimulus sizes. In particular, the tuning curves constituting the “true data” used for the tests in Fig 4 were not part of the training dataset, but were newly generated from the ground-truth model, and should thus be considered held-out test data. Moreover, they included responses to stimuli of sizes that were not covered in the tuning curves used in training.

Fig 4C and 4D provide comparisons of the distributions of these tuning curve attributes under the trained and ground truth SSN models. As in Experiment 1, we measured the mismatch of these distributions using the Kolmogorov-Smirnov (KS) distance. The KS distance for all distributions becomes very small as a result of learning (Fig 4B), reflecting the close fit of all test statistics from the trained model with the data (Fig 4C and 4D). (Note that since the generator was not directly trained to minimize any of the above KS distances, there is no reason why they should decrease monotonically during learning.)

Although moment matching can capture the overall shape of the one-dimensional (marginal) distributions of some of the test statistics (*e*.*g*., see Fig 4D for preferred size), it fails to capture some details in higher-dimensional joint distributions. For example, moment matching underestimates the density of the joint distribution of the suppression index and peak rate between the two peaks. By contrast, the WGAN faithfully captures this joint distribution (Fig 4C). We emphasize again that such summary statistics (and their distributions) did not directly play a role in the WGAN fit; instead, as discussed in subsection “Generative Adversarial Networks” above, the WGAN’s discriminator network automatically discovers the relevant optimal statistic, and in this way fits the full high-dimensional tuning curve distribution, and in particular the low-dimensional distributions plotted in Fig 4C and 4D.

### Experiment 3: Parameter identification

In the point of view adopted in this paper, the distribution of neural tuning curves serves as a probe into the network structure; the richer the tuning curve dataset, the stronger the probe. Richness of tuning curve data can correspond to at least two different factors. One factor is the richness of stimuli, or the breadth and dimensionality of the region of stimulus parameter space covered in the tuning curves. A second factor is the degree to which each neuron’s tuning curve is associated with other functional or anatomical information such as cell type, preferred stimulus, topographic location, etc.

When the probe is not sufficiently strong, it may not serve to fully uncover the network structure as encoded in model parameters. When a model class is at all capable of capturing the observed tuning curve distribution, it may be the case that models within that class but with widely different parameters are equally capable of fitting the tuning curve distribution. In such a scenario the model parameters will not be uniquely identifiable using the available tuning curve data. For example, in Experiment 2 we were able to train an SSN to accurately capture the size tuning curve distribution. However, in many runs the parameters of the trained SSN failed to match the parameters of the ground-truth SSN that had generated the training dataset. This failure is, however, not a failure of the WGAN training algorithm, as the training was consistently successful in capturing the size tuning curve distribution very well. The failure is partly due to the relative poverty of the tuning curve data used. In that example, the dataset contained only the size tuning curves of excitatory and centered neurons (neurons with receptive field or topographic location at the center of the stimulus). Moreover, stimulus parameters other than size, such as contrast, were not varied in the tuning curves. We thus set out to investigate whether enlarging the tuning curve data along some of the mentioned lines can allow for accurate identification of the network parameters using the GAN methodology.

We experimented with including identified inhibitory cell tuning curves in the training data, as well as size tuning curves for offset cells with topographic location not at stimulus center. However, we found that only including offset tuning curves was sufficient to enable parameter identification. We will report the results of this experiment here; in this training dataset we included the size tuning curves of neurons with the following possible offsets from stimulus center: ${\left(x_{i_{p}}\right)}_{p=1}^{O}=\left(0,1/16,1/8,1/4,3/8\right)$.

To fit the SSN to the enriched dataset, here we employed the conditional WGAN (cWGAN) algorithm. As explained in the subsection Conditional GANs above, in cWGAN the discriminator and generator depend on a set of “condition” variables in addition to their inputs in the original WGAN. Here, we provided the topographic offset, $x_{i_{p}}$, of the cells as the conditional input. The cWGAN formalism allows for using tuning curves of neurons for which only some of the offsets are measured. In particular, it allows for exploiting off-center size tuning curves (which are commonly discarded) towards system identification.

**Fig 5:**
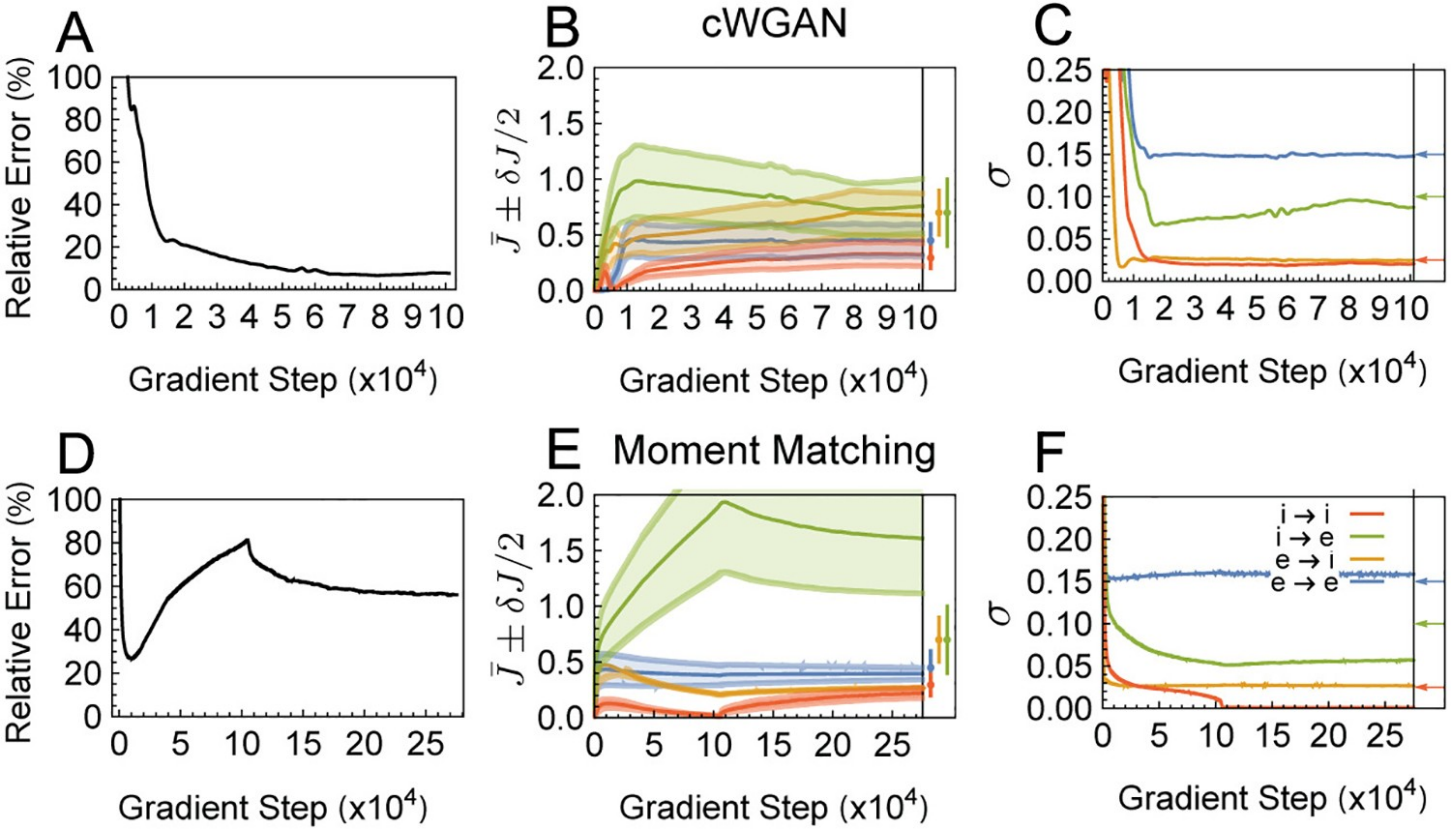
System identification of the SSN model from simulated tuning curve data using the cWGAN method and moment matching methods. Top and bottom rows show the results of cWGAN and moment matching fits, respectively. **(A) & (D)**: symmetric mean average percent error (sMAPE), Eq (44), of all parameters throughout training. **(B) & (E)**: the ${\bar{J}}_{ab}$ (solid lines) and *δJ*_*ab*_ (shaded areas) parameters throughout training. The box to the right shows the true parameters as points (${\bar{J}}_{ab}$) with error bars (*δJ*_*ab*_). **(C) & (F)**: *σ*_*ab*_ throughout training. Arrows to the right of the plot denote the true values of *σ*_*ab*_; note that the *E* → *I* and *I* → *I* arrows occlude each other in panels C and F. In B-C and E-F colors represent connection-types as shown in the legend of B. The input heterogeneity parameter *V* (not plotted) converged to 0.100 for cWGAN, and to 0.116 for moment matching, compared to the value *V* = 0.1.

We compare the quality of fits using the cWGAN and moment matching respectively (Fig 5). Although moment matching produces a reasonable fit to the distribution of tuning curves for the individual features we explored (Fig 4B), the GAN approach significantly outperforms moment matching at parameter identification (Fig 5). This is summarized in the relative error plots in Fig 5A and 5D; relative error was measured using the symmetric mean absolute percentage error (sMAPE), defined in Eq (44) of Materials and methods. In particular, moment matching severely misestimates the *δJ*_*ab*_’s (see Fig 5E), which control the heterogeneity in recurrent horizontal connections. On the other hand, cWGAN was successful at identifying parameters with less than 10% error, and the fit of the summary statistic distributions was excellent.

**Fig 6:**
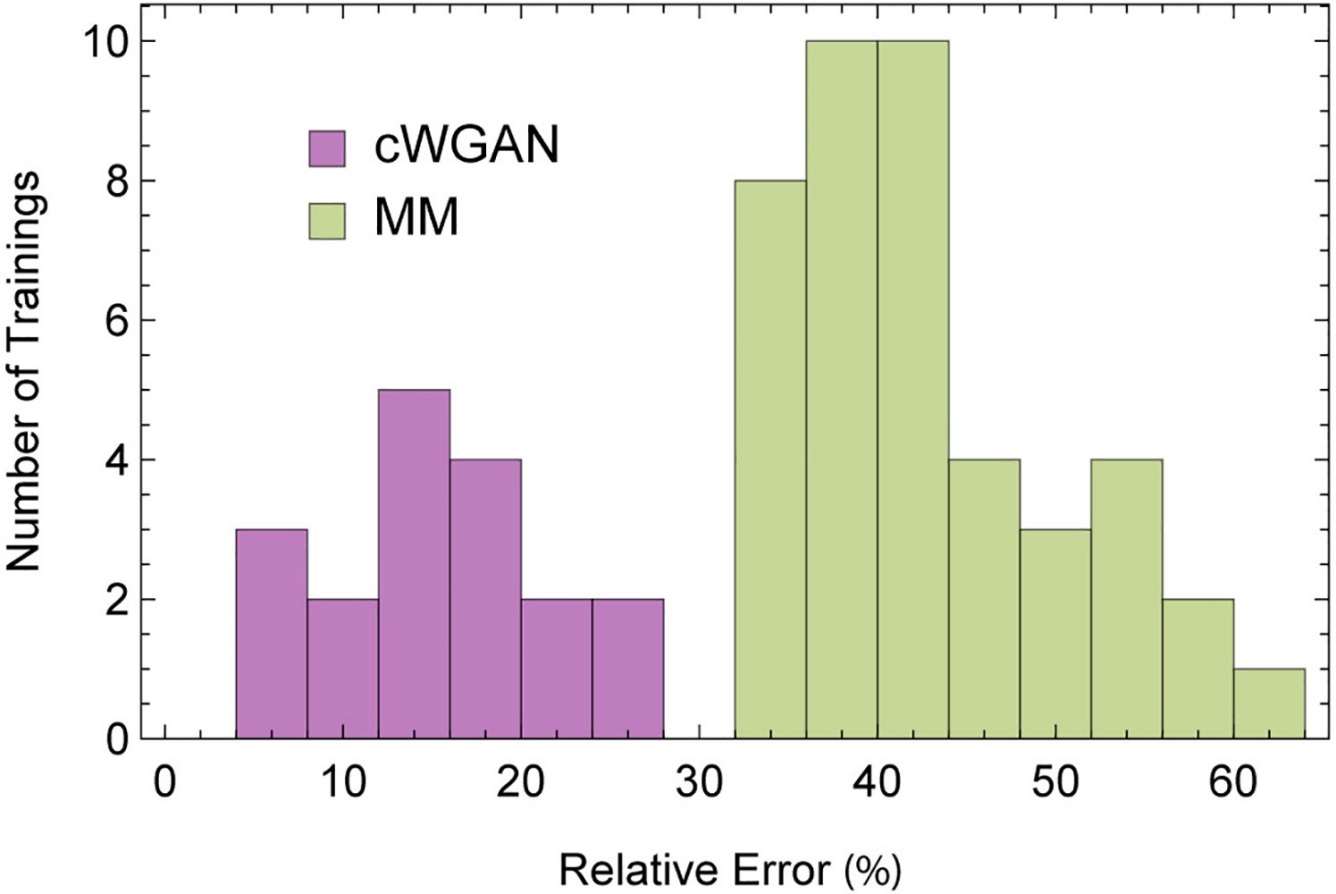
Comparison of conditional Wasserstein GAN and moment matching. Distribution of the relative error (symmetric mean average percent error; sMAPE) for our proposed method (cWGAN, in purple) and moment matching (MM, in Green) across different hyperparameters (see Materials and methods). The bars denote the number of trainings, possibly with different hyperparameters, resulting in an sMAPE within the corresponding bin. cWGAN consistently produced lower estimation errors compared to moment matching.

The results demonstrated in Fig 5 are robust. Fig 6 shows a histogram of percent error (quantified by sMAPE, Eq (44)) for multiple moment matching and cWGAN fits, performed using a wide range of hyperparameters (see Materials and methods, under “Hyperparameters for parameter identification experiment”), including different learning rates for the generator, and for the discriminator in the cWGAN case. To provide a fair comparison of performance between the two methods, in Fig 6 we used an impartial termination criterion which is agnostic to the hyperparameters (see Materials and methods, under “Stopping criterion and performance metric”), for both cWGAN and moment matching. In all cases the cWGAN outperforms moment matching.

We observed that successful cWGAN fits required particularly small generator learning rates. By contrast, in other examples (not reported) in which the tuning curve dataset was richer, and inhibitory neuron tuning curves were also observed by the discriminator, trainings with ten times larger generator learning rates successfully identified the parameters. We also observed that moment matching was able to identify the parameters in those easier cases. More generally, we speculate that harder problems (*i*.*e*., those with poorer training data), such as in Fig 6, may require a smaller WGAN generator learning rate for accurate parameter identification.

## Discussion

Developing biologically grounded mechanistic models that can capture the diversity and heterogeneity of neural response properties and selectivities is an important aim of theoretical neuroscience. Methods that give us the ability to quantitatively constrain such models directly using neural data, such as datasets of tuning curves from a given brain area, can greatly facilitate this pursuit. Such methods allow inferring the structure of biological circuits of interest from observations of neural responses, as encoded, *e*.*g*., by tuning curve distributions.

The statistical fitting of most interesting mechanistic models of brain circuitry using classical likelihood-based approaches is often intractable. A practical solution used in the past has been to instead use models that are designed primarily based on the tractability of their likelihood functions and ease of fitting, as opposed to biological realism or purely theoretical criteria. Therefore the elements and parameters of such models often may not have a direct mechanistic, biological interpretation. Another approach to the challenge of fitting theoretically grounded mechanistic models has been to forego full probabilistic inference using likelihood-based approaches, and use moment matching to only match a few summary statistics (characterizing tuning curves and thus neural responses) between model and data. The drawback of this approach is that it is not information theoretically efficient and does not exploit all the available information in the dataset for the purpose of inferring network properties.

Here, we demonstrated that Generative Adversarial Networks (GANs) enable the fitting of mechanistic models developed in theoretical neuroscience to the *full* joint distribution of neural response data. Conceptually, we proposed to view mechanistic network models with randomness in their connectivity structure or other biophysical parameters as generative models for response tuning curves. Given this formulation, one can exploit a whole suite of recently developed methods in machine learning (of which GANs are an example) for fitting the full output distribution of *implicit* generative models (*i*.*e*., generative models for which the likelihood function is not available or is highly intractable).

In this paper we specifically focused on using the Wasserstein GAN (WGAN) [25] approach for this purpose. In subsection “Generative Adversarial Networks” of Results we reviewed the basic GAN setup and the WGAN algorithm, and conceptually contrasted it with moment matching. In Experiment 1 and 2 we used this method to successfully fit two representative example models from theoretical neuroscience to real or simulated tuning curve data, demonstrating that this technique is applicable to inference based on a wide range of network models, including feedforward and recurrent models of cortical networks. Furthermore, in Experiment 3 we demonstrated that our method is able to consistently and accurately identify the true parameters of a cortical network model using tuning curve data. This experiment moreover provides an example application in which the WGAN approach, which exploits the full tuning curve distribution, is superior to moment matching which only considers a few moments of that distribution. However, we note that even though moment matching is information-theoretically weaker than GANs, in practice it may be advantageous for fitting simple circuit models as it can be trained faster.

In the rest of the Discussion, we review some of the potentials and pitfalls of our approach and some differences with other usages of GANs, in an attempt to make the path clearer for other future applications of and improvement to this approach.

### Conditional *vs*. non-conditional GANs

In our experiments we used both conditional and non-conditional GANs. When using a non-conditional GAN as in Experiments 1 and 2, the discriminator only receives as input the discretized tuning curve in the form $x\equiv {\left(\bar{r}\left(s\right)\right)}_{s=1}^{S}$. Here, the index *s* denotes the stimulus condition, corresponding to combinations of different stimulus parameter values. In this formulation of the tuning curve, the relationship between stimulus parameters and the index *s* is thus lost to the discriminator. In particular, the discriminator is blind to the metric or similarity structure in the stimulus parameter space (*e*.*g*., it does not explicitly know whether two different components of **x** encode responses to very similar or widely different stimuli), and therefore cannot directly exploit that structure in discriminating between true and generated tuning curves. Another drawback of this formulation is that all neurons in the dataset must have been recorded in all stimulus conditions; when there are several stimulus parameters, however, recording each neuron in all conditions for many trials becomes experimentally prohibitive. On the other hand, non-conditional GANs are advantageous in allowing the generator to learn the joint distribution of single-neuron responses across the entire stimulus parameter space; in particular, it enables to fit the marginal distribution of “global” tuning curve features that depend jointly on responses at different values of multiple stimulus parameters.

The conditional GAN approach provides a complementary scheme for describing the tuning curve, *i*.*e*., the relationship between neuronal responses and stimulus parameters. In this case, different values of a subset of stimulus parameters are implicitly represented by the different components of **x**. However, **x** (and its component responses) depend also on another subset of stimulus parameters that are provided explicitly as inputs to the discriminator (as well as the generator), in the form of conditional GAN’s condition variables *c* (more generally, *c* need not be limited to subsets of stimulus parameters, and can, *e*.*g*., also denote a neuron’s cell type or preferred stimulus parameters). Since the value of these parameters is directly provided to the discriminator, the latter is not entirely blind to stimulus similarity structure. On the other hand, the conditional GAN framework only fits the conditional distributions of **x** at different values of *c*. This is beneficial in that we now do not need to record each neuron across all values of *c*. However, by the same virtue, the framework is blind to correlations of single-cell responses at different values of *c* across neurons, and may not fit the distribution of single-neuron tuning curve features that depend jointly on responses to stimuli with different *c*-values. By allowing a trade-off between capturing the joint distribution of single-neuron responses across the entire parameter spaces *vs*. handling a heterogeneous dataset with missing data, the conditional GAN provides additional flexibility in fitting theoretical models to diverse neuronal data.

### Optimization difficulties, underfitting and overfitting

As with any gradient-based training method, it is possible for a GAN to become stuck in suboptimal local minima for the generator (or the discriminator). It is further an open question whether GAN training will always converge [29, 41, 42]. As research in GANs and non-convex optimization advances this issue will be improved. For now avoiding this pitfall will be a matter of the user judging the quality of fit after the fit has reasonably converged. Starting the gradient descent algorithm with several different initial conditions for generator parameters can also help, with some initializations leading to better final fits.

Apart from the above general problems, when the generator is a recurrent neural network (RNN), other problems may arise within each step of gradient descent. When the generator output is based on a steady-state (fixed point) of the RNN, as was the case in our SSN experiment, a potential issue is lack of convergence to a stable fixed point for some choices of recurrent network parameters. In our experiment with SSN, we initialized the generator network in such a way that it initially had a stable fixed point for almost all realizations of **z**. For the SSN this would generically be the case when recurrent excitation (which has destabilizing effects) is sufficiently weak. Hence initializations with small *J*_*EE*_ and *δJ*_*EE*_ are good choices. In addition, a relatively large SSN size *N* improves the stability issue because random quenched fluctuations in total incoming synaptic weights are relatively small when the number of presynaptic neurons is large. Thus, for large networks, for a given choice of network parameters, *θ*, either the network converges to a stable fixed point for almost all **z**, or almost never does. To avoid entering parameter regions leading to instability during training, we added the additional regularizing term Eq (32) to the generator loss. We found that the addition of this term is crucial for the success of the algorithm.

An additional problem particular to optimizing GANs is mode collapse (also known as mode dropping), in which some modes of a multi-modal dataset are not represented (or in the worst case only one mode is represented) in the generative model output [29, 43, 44]. Mode collapse is an example of underfitting. The work presented here did not suffer from mode collapse, likely because of the highly structured models employed. Nevertheless, other applications may suffer from the problem of mode collapse. Many approaches have been explored to prevent mode collapse, and we do not give a comprehensive review, but instead cite a selection of interesting approaches. The WGAN itself, which is employed here, is believed to alleviate mode collapse to some degree [25]. Other more sophisticated approaches exist, including the addition of a mutual information maximizing regularizer between model output and the latent variables [45]. One particularly elegant and effective approach is the PacGAN which provides two or more independent, concatenated generator or data samples to the discriminator so that a model suffering from mode collapse will be recognizable from its lack of diversity [46].

Another possible problem is that of overfitting. In the context of generative model training, extreme overfitting corresponds to the generative model approximately memorizing the individual samples in the training data. In our experiments, this would have corresponded to the trained circuit model only generating a finite set of possible tuning curves, namely the samples seen during training. GANs are prone to sample memorization when their generator and discriminator have high complexity or expressivity. For typical circuit models in neuroscience, however, such as the examples we considered here, the output (*e*.*g*., tuning curve) distribution is expected to have a smooth density (in contrast to a discrete distribution with support on or near the training-set tuning curves) almost everywhere in parameter space; such a generator can never memorize a finite sample of tuning curves.

### Identifiability of circuit parameters

In fitting generative models it is possible for models with widely different parameters to result in nearly identical output distributions. In our case, this corresponds to cases in which networks with widely divergent connectivity or single-cell parameters nevertheless generate very similar tuning curve distributions. In such cases it would be impossible to make precise inferences about network parameters (*e*.*g*., connectivity statistics) using tuning curve data. This problem is exacerbated for the moment matching method, which discards information in the data by reducing the tuning curve distribution to a few moments. In comparison, the problem should generally be less severe for the GAN method which tries to fit the entire distribution. Irrespective of the fitting method, however, there is no general reason why the distribution of a relatively low-dimensional output of the model, such as the tuning curve with respect to one stimulus parameter, would provide sufficient information for constraining all circuit parameters. Fortunately there is nothing in our approach that prevents one from applying it to datasets of tuning curves with respect to several stimulus parameters, or tuning curves of multiple cell types. The general expectation is that the higher the dimension of the stimulus parameter space underlying the tuning curves in the training data, the more identifiable the network parameters become.

For example, in the case of our SSN experiments, we first trained the generative model using only tuning curves with respect to stimulus size. In the parameter identification experiment (Experiment 3), we enriched the size tuning curve dataset by adding center-offset neurons, and additional sampling conditions by adding inhibitory cell tuning curves. The result was an improvement in the robustness and accuracy of model parameter identification. With datasets of sufficiently rich tuning curves, the GAN-based method provides a promising way to infer biophysical networks parameters, such as those governing connectivity statistics. This also has deep implications for experimental design: the standard approach of using optimal stimuli (in the case of our SSN example, gratings with no center offset), or only focusing on excitatory neurons may produce datasets that are insufficiently rich to allow for model-based inference of all circuit parameters of interest. In particular, a framework like GANs can in principle be used to *design* experiments, *i*.*e*., optimally choose the stimulus conditions and quantities to be recorded, to maximize the identifiability of the parameters of a given model.

### Future directions

The current work can be extended along several directions. In addition to the GAN framework, a suite of other methods have also been developed recently in machine learning for fitting generative models [23]. Examples include variational autoencoders [22, 47], and hierarchical and deep implicit models [24]. These methods can also be fruitful for fitting circuit models from neuroscience and inferring circuit parameters. Recent progress in unifying these approaches [48–50] can further inform their future applications. Of special note is the Bayesian methodology of Ref. [24] which in addition to point estimates for parameters, also yields estimates of their posterior uncertainty (or more generally, their approximate posterior distribution).

In the current study, we took the output of the mechanistic circuit model to be tuning curves composed of single-cell trial-averaged sustained responses. But the conceptual framework explained in the beginning of Results and the GAN methodology can also be used to constrain mechanistic network models using data featuring the temporal dynamics of neural activity and higher-order statistics of trial-to-trial variability. For example, the network model can be fit not only to match the distribution of tuning curves encoding trial-averaged single-cell sustained responses, but rather the entire peristimulus time histogram or also the distribution of noise correlations between cell pairs in some cortical area, extracted from simultaneously recorded neural activity.

Lastly, although we focus here on applications to neuroscience and neuronal networks, the proposed framework can potentially serve to fit mechanistic models from other corners of biology, in order to infer the structure of other kinds of biological networks from functional data. For example, the framework can potentially be used to infer the structure of gene regulatory networks from data on the expressions of one or a few genes in different environments.

## Materials and methods

### Conditional Generative Adversarial Networks

Here we provide the complete expressions for the loss functions used in the conditional WGAN (cWGAN) method and a pseudocode for this algorithm. The non-conditional WGAN setup was described in subsection Generative Adversarial Networks of Results. cWGAN’s are similarly composed of a generator and a discriminator, but now both the descriminator $D_{w}$ and generator *G*_***θ***_ depend on a “condition” argument or input, *c*, in addition to their primary inputs [33]. The condition variable *c* can be discrete or continuous and can range over a set of possibilities *C*. When there is only one possibility for *c* (in which case this argument can be dropped) we recover the original (non-conditional) WGAN.

The discriminator and generator loss functions for cWGAN are given by

$${\text{Loss}}_{D}\left(w,\theta \right)=E_{z,c}\left[D_{w}\left(G_{\theta }\left(z;c\right);c\right)\right]-E_{x,c}\left[D_{w}\left(x;c\right)\right]+\left(\text{Gradient}\;\text{Penalty}\right)$$ (8)

$${\text{Loss}}_{G}\left(w,\theta \right)=-E_{z,c}\left[D_{w}\left(G_{\theta }\left(z;c\right);c\right)\right]+{\text{Penalty}}_{G}\left(\theta \right),$$ (9)

respectively. Here $E_{x,c}$ denote the average over a batch of data samples, **x**, together with the conditions, *c*, at which they were recorded. Similarly, $E_{z,c}$ denotes averaging over a batch of noise variables **z**, sampled from their fixed distribution, and conditions *c*, sampled from their empirical distribution. Note that **z** and *c* are treated as independent random variables. The “Gradient Penalty” term forces the gradient of $D_{w}$ (with respect to its first argument) to be close to one; following the recipe of Ref. [26] we set it to

$$\begin{array}{c} \text{Gradient}\;\text{Penalty}=\lambda E_{z,x,c,\epsilon }[(\|\nabla D_{w}\left(\epsilon x+\left(1-\epsilon \right)G_{\theta }\left(z;c\right);c\right){\|}_{2}-1{)}^{2}] \end{array}$$ (10)

where *ϵ* is a random variable with uniform distribution on [0, 1], and the gradient ∇ is taken with respect to the first argument of $D_{w}$, and not the condition *c* or parameters **w**. Finally, the term Penalty_*G*_(***θ***) in the generator loss denotes possible generator model dependent regularization terms. We did not include any such term for the feedforward network example presented in Experiment 1. The Penalty_*G*_(***θ***) for the recurrent SSN example is described in Eq (32) of Materials and methods.

A stochastic gradient descent algorithm for cWGAN based on these loss functions is shown in Algorithm 1.

**Algorithm 1**: Improved cWGAN algorithm based on Ref. [26]. For all models, we use $n_{D}=5$. For update method, we either use Adam with *β*_1_ = 0.5, *β*_2_ = 0.9, *ϵ* = 10^−8^ [26] or RMSProp with *ρ* = 0.9, *ϵ* = 10^−6^. For the feedforward model, we use $\alpha_{D}=\alpha_{G}=0.001$, *m* = 30, *γ* = 0, Penalty_*G*_(***θ***) = 0, and ${\text{Update}}_{D}={\text{Update}}_{G}=\text{Adam}$. The generating processes are considered normal always and the test^†^ always passes. For the SSN $\alpha_{D}=0.02$, *α*_*G*_ = 10^−4^, *m* = 128, *γ* = 0.001, Penalty_*G*_(***θ***) = Eq (32). The discriminator is updated only if generating processes (*G*_***θ***_(**z**)) pass a test^†^ for “normality” (see Eq (33)). We use always RMSProp for the generator in the SSN experiments. We use RMSProp for the discriminator in Fig 4, Adam in Fig 5, and aggregate the results with both RMSProp and Adam in Fig 6.

**Input**: data distribution $P_{r}$, the gradient penalty coefficient λ, the number of discriminator iterations per generator iteration $n_{D}$, the batch size *m*, update methods ${\text{Update}}_{D}\left(\cdot \right)$, Update_*G*_(·), learning rates $\alpha_{D}$, *α*_*G*_, weight decay hyperparameter *γ*, and the initial discriminator ***w***_0_ and generator ***θ***_0_ parameters.

***θ*** ← ***θ***_0_;

***w*** ← ***w***_0_;

**while *θ*** *has not converged* **do**

 **repeat**

  **for** *i* = 1, …, *m* **do**

   Sample real data $\left(x,c\right)∼P_{r}$, latent variable **z** ∼ *p*(**z**), a random number *ϵ* ∼ *U*[0, 1].;

   $\tilde{x}\leftarrow G_{\theta }\left(z;c\right)$;

   $\hat{x}\leftarrow \epsilon x+\left(1-\epsilon \right)\tilde{x}$;

   ${\text{Loss}}_{D}^{\left(i\right)}\leftarrow D_{w}\left(\tilde{x};c\right)-D_{w}\left(x;c\right)+\lambda (\|\nabla_{\hat{x}}D_{w}\left(\hat{x};c\right){{\|}_{2}-1)}^{2}$;

  **end**

  **if** *generating processes are normal*^†^ **then**

   $w\leftarrow {\text{Update}}_{D}\left(\nabla_{w}\frac{1}{m}\sum_{i=1}^{m}{\text{Loss}}_{D}^{\left(i\right)},w,\alpha_{D}\right)-\gamma w$;

  **end**

 **until** $n_{D}$ *updates are tried*;

 Sample latent variables ${\left(z^{\left(i\right)}\right)}_{i=1}^{m}∼p\left(z\right)$ for a mini-batch.;

 Sample conditions from real data ${\left(\cdot ,c^{\left(i\right)}\right)}_{i=1}^{m}∼P_{r}$ for a mini-batch.;

 $\theta \leftarrow {\text{Update}}_{G}(-\nabla_{\theta }(\frac{1}{m}\sum_{i=1}^{m}D_{w}\left(G_{\theta }\left(z^{\left(i\right)};c^{\left(i\right)}\right)\right)+{\text{Penalty}}_{G}\left(\theta \right)),\theta ,\alpha_{G})$;

**end**

### Alternatives to WGAN, and alternative views of GANs

In this subsection, we provide a quick review of some of the alternatives to the WGAN loss functions, Eqs (3) and (4), developed in the GAN literature, which can be useful in computational biology applications (for a more comprehensive review of GANs see [29]). We also point out an alternative view of GANs inspired by the energy-based framework for unsupervised learning [51, 52].

The original GAN developed in [23] was framed as a minimax or zero-sum game in which the generator and discriminator competed by respectively minimizing and maximizing the same loss:

$${\text{Loss}}_{G}\left(w,\theta \right)=-{\text{Loss}}_{D}\left(w,\theta \right)=E_{x}\left[\text{log}\;D_{w}\left(x\right)\right]+E_{z}\left[\text{log}\left(1-D_{w}\left(G_{\theta }\left(z\right)\right)\right)\right].$$ (11)

Given this form for the loss, the discriminator, $D_{w}\left(x\right)$, can be thought of as a binary classifier that estimates the conditional probability of the true or empirical category (*vs*. “fake” or *G*-generated category) given the observation **x**. In this case, for a fixed generator whose output has distribution *P*_***θ***_(**x**), the theoretically optimal discriminator is given by $D_{*}\left(x\right)=\frac{P_{\text{true}}\left(x\right)}{P_{\text{true}}\left(x\right)+P_{\theta }\left(x\right)}$, where *P*_true_(**x**) is the true data distribution or density. In that sense, a sufficiently expressive and well-trained discriminator (using Eq (11)) learns the ratio of the model likelihood, *P*_***θ***_(**x**), to the true data density.

Moreover, for this optimal discriminator solution the loss Eq (11) reduces to the Jensen-Shannon (JS) divergence (up to additive and multiplicative constants) between *P*_true_(**x**) and *P*_***θ***_(**x**) [29]. Thus the generator is theoretically trained to minimize the JS distance. By comparison, as noted in subsection “Generative Adversarial Networks” of Results, WGANs theoretically minimize the Wasserstein or earth-mover’s distance between *P*_true_(**x**) and *P*_***θ***_(**x**).

Both of these divergence or distance measures are in contrast to the Kullback-Leibler (KL) divergence *D*_*KL*_(*P*_true_‖*P_θ_*) which is effectively minimized in classical maximum-likelihood estimation of the generator (or equivalently, of its parameters ***θ***). Correspondingly, others have modified the generator-loss so that, given the theoretically optimal discriminator, it reduces to the KL divergence, or other divergences [53–56]. For example, to minimize the KL divergence, [56] used the same discriminator loss as in Eq (11), in conjunction with the modified generator loss

$$\begin{array}{c} {\text{Loss}}_{G}\left(w,\theta \right)=E_{z}\left[f\left(D_{w}\left(G_{\theta }\left(z\right)\right)\right)\right] \end{array}$$ (12)

where $f\left(u\right)=\frac{u}{1-u}\text{exp}\left(\frac{u}{1-u}\right)$.

As we mentioned in the Discussion, Ref. [24] developed a GAN-like framework for variational Bayesian estimation of implicit generative models, in which a discriminator-like network was trained to estimate the generator’s likelihood function. However, this Bayesian framework goes beyond maximum-likelihood estimation: Ref. [24] developed a three-network framework in which, in addition to the (implicit) generative model and discriminator (which estimates the generator’s likelihood), a third network is trained to provide an approximation to the Bayesian posterior over ***θ*** (as in more general variational Bayesian approaches [22, 57]).

Finally, we mention energy-based GANs (EBGANs), which are inspired by the energy-based framework for learning [51, 52]. EBGANs use the following loss functions for the generator and the discriminator:

$${\text{Loss}}_{D}^{\text{EBGAN}}\left(w,\theta \right)=E_{x}\left[D_{w}\left(x\right)\right]+E_{z}\left[{\left(m-D_{w}\left(G_{\theta }\left(z\right)\right)\right)}_{+}\right]$$ (13)

$${\text{Loss}}_{G}^{\text{EBGAN}}\left(w,\theta \right)=E_{z}\left[D_{w}\left(G_{\theta }\left(z\right)\right)\right]$$ (14)

where (*x*)_+_ = max(0, *x*) denotes rectification, *m* > 0 is a positive margin parameter, and $D_{w}$ is constrained to be non-negative (see [58]).

EBGANs are in part motivated by an alternative view of the role of the discriminator in GANs. In the viewpoint expressed in subsection “Generative Adversarial Networks” of Results, the discriminator is thought of as a flexible and trainable objective function that is used for training the generator. In this interpretation, the generator is key and the discriminator is auxiliary. However, an alternative viewpoint is also possible in which the discriminator is key and the generator is auxiliary (see appendix B of [58]). In this view, which is suggested by the energy-based framework for learning [51, 52], the discriminator learns the relative density of the data distribution. More precisely, it is thought of as an energy function that is shaped during training to be low in regions of high data density, and high elsewhere. This is achieved by minimizing a loss functional during learning; Eq (13) is an example of such a loss functional. Imagine for simplicity that true data lie on a subspace or manifold in the data space. The loss functional is designed such that true data samples serve to “push down” the energy function (*i*.*e*., increase the probability density) on the data manifold, while another mechanism is used to “pull-up” the energy function (*i*.*e*., reduce the probability density) outside that manifold. In simple unsupervised learning methods such as principle component analysis the pull-up of energy is implicitly achieved due to the rigidity of the energy function itself (see [52]). But when the energy function is sufficiently flexible, an explicit term in the loss functional is needed to pull it up outside the true data manifold. Fake or simulated data points, referred to as contrastive samples, can be used for energy pull-up. In Eq (13), the first and second term serve to push up and pull down the energy function, $D_{w}$, respectively. Accordingly, in the alternative viewpoint of GANs, the generator is viewed as merely providing such contrastive samples to the discriminator.

As explained below, in contrast to many applications of unsupervised learning, the generator is indeed central in our intended applications, and we therefore focused on the first interpretation of GANs in subsection “Generative Adversarial Networks” of Results. Nevertheless, unsupervised learning of generative models is often carried out with the end-goal of learning a useful representation of observed data, which can, *e*.*g*., serve to compress or reduce the dimensionality of data. In our case this corresponds to dimensionality reduction of tuning curves. Estimating the density of observed data is a related end-goal of unsupervised learning, which can, *e*.*g*., serve data restoration (*e*.*g*., image denoising) applications. Even though these were not the applications motivating the current study (see the next subsection), they do constitute potential applications of GANs in computational biology. The alternative view of the role of discriminator can be more advantageous in such settings.

### Differences with common machine learning applications

In most applications of GANs in machine learning and artificial intelligence, the generative model is an artificial neural network (*e*.*g*., a deconvolutional deep feedforward network), and that network’s individual connection weights constitute the generator’s trainable parameters, ***θ***. In most such applications, these parameters are not objects of interest on their own and may not be mechanistically meaningful or interpretable. Similarly, the development of generative models in such domains is not necessarily concerned with capturing the true, physical mechanisms underlying the modeled data. There and in other unsupervised learning settings, the end goal is to achieve a generator that produces realistic data objects (*e*.*g*., images), or to accurately estimate the data density or its support (which can serve for dimensionality reduction or denoising of data). In such domains, the discriminator itself may be of central importance as it can potentially estimate the relative density of data objects (see the previous subsection for a discussion of this viewpoint).

By contrast, in applications of primary interest to us, it is the generator that is of primary interest. The core of the generator is a circuit model, developed independently of the method used to fit it (in our case GANs), with the scientific goal of uncovering the circuit mechanisms in a neural or biological system. In particular, in our applications, the generator parameters ***θ*** typically correspond to physiological and anatomical parameters with clear mechanistic interpretations. Correspondingly, the circuit model is highly structured, with that structure strongly informed *a priori* by biological domain knowledge and the scientific desire for parsimony. This allows for post-training tests of the model that go beyond testing the fit to held-out data of the same type as training data, and may not be conceivable in many machine learning applications. For example, one can imagine training an SSN circuit model on size-tuning curves (as we did in Experiment 1–3) but test it using stimuli with varying strengths or contrasts and compare the generated distribution of contrast-response function against data. Alternatively, one can feed the trained SSN dynamical noise and then compare the statistics of temporal neural variability (such as pairwise noise correlations) against empirical data. In such tests of generalization, the data-space itself changes (and not just the data points in it) between training and testing, and therefore a discriminator trained on one space simply cannot generalize to the other; the link between the two data-spaces is solely provided by the generative mechanistic circuit model. The promise of a faithful scientific model of a brain network is, in principle, to capture all such neural data at least approximately; the above scenarios can thus be used as strong tests for such models. To perform such tests faithfully and quantitatively, a strong fitting procedure is necessary.

### Feedforward model of M1

The model of primary motor cortex (M1) tuning curves proposed by Ref. [4] is a two-layer feedforward network, with an input layer, putatively corresponding to the parietal reach area or to premotor cortex, and an output layer corresponding to M1 (see Fig 2). Ref. [4] introduced their model in two versions, a basic one, and an “extended” version. We have used their enhanced version with small modifications noted in Experiment 1 and below which allow our approach to be used. In particular, we did not model response selectivity to hand posture (supination or pronation), and ignored that label in the dataset; *i*.*e*., we blindly mixed hand-position tuning curves across pronation and supination conditions, as if they belonged to different neurons. We further simplified the dataset by removing spatial scale information in the positions of target hand locations, which varied slightly between experimental sessions, by rescaling the distance between adjacent hand position to be 1. We randomly selected half of the hand-position tuning curves to be our training dataset, and used the other half as held-out data to evaluate the goodness of model fits (presented in Fig 3).

The input to the feedforward network is the 3D hand position **x**_*s*_, with *s* ∈ {1, ⋯, 27} indexing the 3 × 3 × 3 grid of possible target locations. The input layer neurons have Gaussian receptive fields defined on the 3D hand position space. The activation, *h*_*i*_(*s*) of neuron *i* in the input layer with receptive field centered at **x**_*i*_ is thus $h_{i}\left(s\right)\propto \text{exp}\;(-\frac{1}{2\sigma_{i}^{2}}{\left\|x_{s}-{\bar{x}}_{i}\right\|}^{2})$ in condition *s* (when the hand is at **x**_*s*_). Across the input layer, the Gaussian centers **x**_*i*_ form a fine cubic grid that interpolates and extends (by 3 times) the 3 × 3 × 3 stimulus grid along each dimension. Whereas Lalazar et al. [4] used a grid with 100 points along each axis, we reduced this to 40 to allow faster computations (we checked that changing this resolution beyond 40 only weakly affects the results). Across the input layer, the receptive field widths, *σ*_*i*_, were randomly and independently sampled from the uniform distribution on the range [*σ*_*l*_, *σ*_*l*_ + *δσ*]. This can be expressed by writing $\sigma_{i}=\sigma_{l}+z_{i}^{\sigma }\delta \sigma$ where $z_{i}^{\sigma }$ are uniformly distributed on [0, 1] and independent for different *i*’s across the input layer.

The feedforward connections from the input to output layer are sparse and random, with a connection probability of 0.01. In our implementation of this model, the strength of the nonzero connections were sampled independently from the uniform distribution on the range [0, *J*]. Since output layer neurons are independent, it suffices to describe the model with a single output neuron. If we denote the connections received by this neuron from the *i*-th input layer neuron by $J_{i}$, we can thus write: $J_{i}=JM_{i}z_{i}^{J}$ where $z_{i}^{J}$’s are sampled independently from the standard uniform distribution on [0, 1], and *M*_*i*_ is a binary 0/1 mask that is nonzero with probability 0.01.

The response of the output layer neuron is given by a rectified linear response function with threshold *ϕ*. The threshold *ϕ* was sampled uniformly from the range [*ϕ*_*l*_, *ϕ*_*l*_ + *δϕ*]. Equivalently, *ϕ* = *ϕ*_*l*_ + *z*^*ϕ*^ *δϕ* with *z*^*ϕ*^ a standard uniform random variable.

The model thus has five trainable parameters ***θ*** = (*σ*_*l*_, *δσ*, *J*, *ϕ*_*l*_, *δϕ*) (listed in Table 2). For a choice of ***θ***, the collection of quenched noise variables, $z=(z^{\phi },{\left(z_{i}^{\sigma }\right)}_{i=1}^{40^{3}},{\left(z_{i}^{J}\right)}_{i=1}^{40^{3}},{\left(M_{i}\right)}_{i=1}^{40^{3}})$, fully determine the network structure: all input layer receptive field sizes, individual feedforward connection strengths, and output layer neural thresholds for a particular network realization. The response $\bar{r}\left(s;z,\theta \right)$ of an output neuron in condition *s* (for *s* ∈ {1, ⋯, 27}) is thus given by

$$\begin{array}{c} \bar{r}\left(s;z,\theta \right)=[\sum_{i=1}^{40^{3}}J_{i}\;h_{i}\left(s\right)-\phi_{i}{]}_{+} \end{array}$$ (15)

where

$$h_{i}\left(s\right)=\frac{1}{Z(s)}\text{exp}\;(-\frac{1}{2\sigma_{i}^{2}}{\left\|x_{s}-{\bar{x}}_{i}\right\|}^{2})$$ (16)

$$J_{i}=J\;M_{i}\;z_{i}^{J}$$ (17)

$$\phi =\phi_{l}+z^{\phi }\delta \phi ,$$ (18)

$$\sigma_{i}=\sigma_{l}+z_{i}^{\sigma }\delta \sigma ,$$ (19)

$$z^{\phi },z_{i}^{J},z_{i}^{\sigma }\overset{iid}{∼}\;\;U\left[0,1\right]$$ (20)

$$M_{i}\overset{iid}{∼}\;\;\text{Bern}\left(0.01\right)$$ (21)

where [*u*]_+_ = max(0, *u*) denotes rectification, and *Z*(*s*) is a normalizing factor such that ∑_*i*_ *h*_*i*_(*s*) = 1. Crucially, the network’s output, $G_{\theta }\left(z\right)\equiv {\left(\bar{r}\left(s;z,\theta \right)\right)}_{s=1}^{27}$, is differentiable with respect to each component of ***θ***, we can thus use the output gradient with respect to model parameters to optimize the latter using any variant of the stochastic gradient descent algorithm. Note that Eqs (17)–(19) constitute an example of the “sampler function” *g*_***θ***_(**z**) introduced in the subsection “Mechanistic network models as implicit generative models” of Results (here the vector of synaptic weights $J_{i}$ corresponds to **W**, while the vector (*ϕ*, ***σ***) capturing single-cell properties corresponds to **γ**).

**Table 2:** The description of the parameters of the feedforward model fit to tuning curve data.

Parameter	Description
*σ*_*l*_	Lower bound of receptive field size range
*δσ*	Width of receptive field size range
*J*	Scale of connection strengths
*ϕ*_*l*_	Lower bound of threshold range
*δϕ*	Width of threshold range

### Recurrent SSN model

Here we provide the technical details of the simulations, fit and analysis of the Stabilized Supralinear Network (SSN) model of the experiments in Experiment 1–3. The SSN is a recurrent network of excitatory (*E*) and inhibitory (*I*) neurons. The dynamical state of the network is the vector of firing rates **r**(*t*) of the *N* network neurons. The rate vector is governed by the differential equation

$$\begin{array}{c} \tau \frac{dr}{dt}=-r+f\;(Wr+FI(s)), \end{array}$$ (22)

where *W* and *f* denote the recurrent and feedforward weight matrices (with structure described below), the diagonal matrix $\tau =\text{Diag}\;({\left(\tau_{i}\right)}_{i=1}^{N})$ contains the neural relaxation time constants, *τ*_*i*_, and **I**(*s*) denotes the stimulus input in condition *s*, with *s* ∈ {1, …, *S*}. The key feature of the SSN is the input-output nonlinearity of its neurons, which in the original model is a supralinear rectified power-law function: $f\left(u\right)=k{\left[u\right]}_{+}^{n}$ ([*u*]_+_ = max(0, *u*) and *n* > 1 and *k* > 0 are constants).

During the training of the model inside the fitting algorithm, however, the model may explore non-biological regions in parameter space that may lead to divergence of model firing rates. To tame such divergences and enforce numerical stability during training, we modified the neural input-output nonlinearity in the model as follows. We let *f*(*u*) be a rectified power-law in the biologically relevant range, but smoothly connected it to a saturating branch at very high rates. More precisely we took

$$\begin{array}{c} f\left(u\right)=\{\begin{array}{cc} k{\left[u\right]}_{+}^{n} & \text{if}\;u<u_{0}\\ r_{0}+\left(r_{1}-r_{0}\right)\tanh(n\frac{r_{0}}{r_{1}-r_{0}}\frac{u-u_{0}}{u_{0}}) & \text{otherwise} \end{array} \end{array}$$ (23)

where *k* = 0.01, *n* = 2.2, *r*_0_ = 200 Hz, *r*_1_ = 1000 Hz, and *u*_0_ = (*r*_0_/*k*)^1/*n*^.

In our SSN examples, we chose to have all random structural variability (which is the source of heterogeneity manifesting in tuning curve shapes) occur in the connectivity matrices *W* and *f*. As described in Experiment 2 (see Fig 2), we experimented with SSN models with one-dimensional topographic structure, on which the structure of *W* and *f* depend. The model has a neuron of each type, *E* and *I*, at each topographic spatial location; for *M* topographic locations, the network thus contains *N* = 2*M* neurons. Below, for the *i*-th neuron, we denote its type by *α*(*i*) ∈ {*E*, *I*} and its topographic location by *x*_*i*_. We let *x*_*i*_’s range from −0.5 to 0.5 on a regular grid.

The statistical ensemble for *W* was described in Experiment 2: the random variability of the matrix elements *W*_*ij*_’s was taken to be independent with uniform distribution, with mean and range that depend on the pre- and post-synaptic cell types and topographic distances. More precisely, for each instance of the model we generated *W* via

$$W_{ij}=\varsigma_{b}\;\left(J_{ab}^{<}+z_{ij}\;\delta J_{ab}\right)\;\text{exp}(-\frac{{\left(x_{i}-x_{j}\right)}^{2}}{2\sigma_{ab}^{2}}),\;a=\alpha \left(i\right),b=\alpha \left(j\right)$$ (24)

$$z_{ij}\overset{iid}{∼}U\left[0,1\right]$$ (25)

where *ς*_*b*_ = 1 or −1 if *b* = *E* or *I*, respectively, and *U*[0, 1] denotes the uniform distribution on the interval [0, 1]. Thus the average weight is $\langle W_{ij}\rangle ={\bar{J}}_{ab}\;\text{exp}\left(-{\left(x_{i}-x_{j}\right)}^{2}/\left(2\sigma_{ab}^{2}\right)\right)$, where we defined ${\bar{J}}_{ab}=J_{ab}^{<}+\delta \;J_{ab}/2$, while the standard deviation $\text{SD}\left[W_{ij}\right]=\frac{1}{2\sqrt{3}}\delta J_{ab}\;\text{exp}\left(-{\left(x_{i}-x_{j}\right)}^{2}/\left(2\sigma_{ab}^{2}\right)\right)$. All parameters, $J_{ab}^{<}$, *δJ*_*ab*_, *σ*_*ab*_ were constrained to be non-negative (which is equivalent to the constraints ${\bar{J}}_{ab}\geq \delta J_{ab}/2\geq 0$ and *σ*_*ab*_ ≥ 0, for the alternative parameterization using ${\bar{J}}_{ab}$, *δJ*_*ab*_, *σ*_*ab*_). The first two constraints (together with the sign variable *ς*_*b*_ in Eq (24)) ensure that any realization of *W*_*ij*_ satisfies Dale’s principle [39, 40].

We chose the feedforward weight matrix, *f*, to be diagonal with weights having independent random heterogeneity across network neurons. More precisely, for a network of *N* neurons, *f* was an *N* × *N* diagonal matrix generated via

$$F=\text{Diag}({\left(1+z_{i}^{F}V\right)}_{i=1}^{N})$$ (26)

$$z_{i}^{F}\overset{iid}{∼}U\left(\left[-1,1\right]\right)$$ (27)

where the binary random variables $z_{i}^{F}$ are sampled independently and uniformly from [−1, 1].

Our recurrent and feedforward connectivity ensemble is thus characterized by 13 non-negative parameters (enumerated in Table 3): the parameter *V* that controls the degree of disordered heterogeneity in feedforward weights, as well as the elements of the three 2 × 2 matrices $J_{ab}^{<}$, *δJ*_*ab*_, *σ*_*ab*_ (where *a*, *b* ∈ {*E*, *I*}), which control the average strength, disordered heterogenetity, and spatial range of recurrent horizontal connections. These constituted the parameters

$$\begin{array}{c} \theta ={\left(J_{ab}^{<},\;\delta J_{ab},\;\sigma_{ab},V\right)}_{a,b\in \{E,I\}}, \end{array}$$ (28)

that were fit using the WGAN (or moment matching). (Because enforcing the non-negativity constraints on the set ${\left(J_{ab}^{<},\;\delta J_{ab},\;\sigma_{ab},V\right)}_{a,b\in \{E,I\}}$ is easier than enforcing the constraints on the set ${\left({\bar{J}}_{ab},\;\delta J_{ab},\;\sigma_{ab},V\right)}_{a,b\in \{E,I\}}$ introduced in Experiment 2, we used the parametrization of Eq (28) in our WGAN implementation.) While these parameters described the statistics of connectivity, a specific realization of the network is determined by the high-dimensional fixed-distribution random variables of the GAN formalism, **z**, in addition to ***θ***. The former is composed of the *N*^2^ independent, standard uniform random variables ${\left(z_{ij}\right)}_{i,j=1}^{N}$, and the *N* independent random variables ${\left(z_{i}^{F}\right)}_{i=1}^{N}$ which are sampled uniformly from [−1, 1]. Also note that Eqs (24) and (26) constitute an example of the sampler function, *g*_***θ***_ (**z**), introduced in the subsection “Mechanistic network models as implicit generative models” of Results.

**Table 3:** The description of the parameters of the SSN inferred from tuning curve data (*a*, *b* ∈ {*E*, *I*}).

Parameter	Description
*σ*_*ab*_	connection length scales
*J*_*ab*_	lower bounds of connection strengths
*δJ*_*ab*_	widths of connection strength distribution
*V*	randomness of the stimulus amplitude

In our example experiments, we simulated the fitting of an SSN model of V1 to datasets of stimulus size tuning curves of V1 neurons [8]. As a simple model of the visual input to V1 evoked by a grating of diameter *b*, the stimulus input to neuron *i* was modeled as

$$\begin{array}{c} I_{i}\left(b\right)=A\;\sigma \;(l^{-1}\left(b/2+x_{i}\right))\sigma \;(l^{-1}\left(b/2-x_{i}\right)), \end{array}$$ (29)

where *σ*(*u*) = (1 + exp(−*u*))^−1^ is the logistic function, *A* denotes the stimulus strength or contrast. Thus, the stimulus targets a central band of width *b* centered on the middle of the topographic grid (see Fig 2B). The parameter *l* determines the smoothing of the edges of the stimulated region. For training the model, we chose the sizes from a set of *S* = 8 different sizes (0, 1/16, 1/8, 3/16, 1/4, 1/2, 3/4, 1) (measured in units of the total length of the network). Letting *b*_*s*_ denote the size in stimulus condition *s* (*s* ∈ {1, ⋯, *S*}), the **I**(*s*) of Eq (22) and Experiment 2 is given by **I**(*s*) = **I**(*b*_*s*_), with a slight abuse of notation.

The output of the SSN, considered as a generative model for tuning curves, are the size tuning curves of a subset of network neurons which we call “probe” neurons. We define the tuning curve of these neurons in terms of their sustained responses evoked by different stimuli. Thus given a specific realization of the SSN, for each stimulus *s* ∈ {1, …, *S*}, we first calculate the sustained network response vector by the temporal average between *t*_1_ and *t*_2_

$$\begin{array}{c} \bar{r}\left(s\right)=\frac{1}{T}\sum_{k=1}^{T}r\left(t_{1}+k\;\Delta t\right) \end{array}$$ (30)

where Δ*t* is the Euler integration step and *T* = (*t*_2_ − *t*_1_)/Δ*t*. We choose *t*_1_/Δ*t* = 200 and *t*_2_/Δ*t* = 240 to balance the computational cost and the accuracy for approximating the true steady-state. Given the sustained network response $\bar{r}\left(s\right)$ and an *a priori* selected set of *O* probe neurons with indices **i** = (*i*_1_, *i*_2_, …, *i*_*O*_) (the probe neurons can equivalently be defined by their types and topographic locations), we define the output of the SSN generative model (the GAN generator) to be the vector

$$\begin{array}{c} G_{\theta }\left(z;c=x_{i_{p}}\right)={\left({\bar{r}}_{i_{p}}\left(s\right)\right)}_{s=1}^{S}. \end{array}$$ (31)

Here, $x_{i_{p}}$ denotes the topographic location of the *p*-th probe neuron, and we have now made the dependency of the output on the quenched noise variables, **z**, and model parameters explicit. We treated the probe neuron’s topographic location, $x_{i_{p}}$, as the condition *c* in conditional WGAN (cWGAN). In this paper, we only probed excitatory neurons, *i*.*e*., *α*(*i*_*p*_) = *E*.

In experimental recordings, typically the grating stimulus used to measure a neuron’s size tuning curve is centered on the neuron’s receptive field. To model this stimulus centering, we always set the first probe neuron *i*_1_ to be at the center of the topographic grid (*i*.*e*., $x_{i_{1}}=0$), which was the center of the stimulus. In Experiment 2 we only fit the model to the size tuning curves of “centered” excitatory neurons. Since in that case there was only one probe neuron (or cWGAN condition), we denoted the model output more succinctly by *G*_***θ***_(**z**), dropping its second argument. By contrast, in Experiment 3 we included tuning curves of neurons with offset receptive fields (topographic locations) in the training dataset and employed a conditional WGAN.

Note that tuning curves for networks such as the SSN described here which have partially random connectivity, show variability across neurons as well as across different realizations of the network for a fixed probe neuron. When the network size *N* is large, typically a local self-averaging or “ergodicity” property is expected to emerge: the empirical distribution, in a single network realization, across the tuning curves of neurons of the same type and with nearby topographic locations should approximate the distribution across different **z** for a neuron of pre-assigned index (*i*.*e*., type and location). Although we did not do so in our experiments, one may exploit this ergodicity for more efficient training and testing of the model by sampling multiple nearby sites from each connectivity matrix realization.

Given a dataset of size tuning curves, we would like to find model parameters, $\theta ={\left(J_{ab}^{<},\;\delta J_{ab},\;\sigma_{ab},V\right)}_{a,b\in \{E,I\}}$, that produce a matching model distribution of tuning curves. In this paper we constructed a simulated training dataset of size tuning curves using a “ground truth” SSN model, with parameters ***θ***^truth^ given in Table 1. All other model parameters were the same between the ground truth and trained SSN models, and had the values: *N* = 402, *k* = 0.01, *n* = 2.2, *τ*_*E*_/Δ*t* = 20, *τ*_*I*_/*τ*_*E*_ = 1/2, *A* = 20, *l* = 2^−5^.

During training, according to Algorithm 1, every time *G*_***θ***_ (**z**; *x*) was evaluated, we simulated the trained SSN using the forward Euler method for *t*_2_/Δ*t* = 240 steps (see also Eq (30)). The gradients of the generator output or the generator loss with respect to parameters ***θ*** were calculated by standard back-propagation through time (BPTT). To avoid numerical instability, the parameters $J_{ab}^{<},\;\delta J_{ab},\;\sigma_{ab}$ were clipped at 10^−3^ during training. To exclude extremely large (non-biological) values, we also clipped them below 10. We also clipped *V* to bound it within the interval [0, 1]. (These upper bounds can be thought of as imposed Bayesian priors on these parameters.)

Moreover, during training, the SSN may be pushed to parameter regions in which, for some realizations of the quenched noise variables **z**, the network does not converge to a stable fixed point. Since an implicit model assumption of the SSN is to model sustained responses by stable fixed points of the model with rates in the biological range, dynamical non-convergennce and very high rates can be (strongly) penalized. We encouraged the firing rate of the all SSN neurons to uniformly remain below a permissive threshold of 200 Hz, by adding the following penalty term to the generator loss

$$\begin{array}{c} {\text{Penalty}}_{G}\left(\theta \right)=\frac{\eta }{NTm}\sum_{j=1}^{m}\sum_{k=1}^{T}{\left\|{\left[r\left(t_{1}+k\;\Delta t;z^{\left(j\right)}\right)-200\right]}_{+}\right\|}_{1} \end{array}$$ (32)

where *m* is the size of the mini-batch in the gradient descent, *T* = (*t*_2_ − *t*_1_)/Δ*t* is the time window for calculating the sustained response Eq (30), and *η* = 100 is the weight of the penalty relative to the WGAN generator loss. This penalty, together with the modified neural input-output nonlinearity descrbied in (Eq (23)), ameliorated diverging solutions of SSN dynamics and the resulting extremely large or small generator gradients.

Once the SSN network produces large output rates, it disrupts the learning in the discriminator. Furthermore, Eq (32) alone can fix such behavior of SSN without relying on the discriminator to learn to adapt to new extremely strong inputs (which are the SSN’s output rates). Thus, we find that it is better to skip such generator output samples for stabilizing learning through the GAN framework. Namely, we update the discriminator parameters for a mini-batch only if

$$\begin{array}{c} {\text{Penalty}}_{G}\left(\theta \right)<1 \end{array}$$ (33)

where the bound 1 is rather arbitrary. We observed that for many successful trainings, such large firing rates never occur.

In order to encourage convergence to a fixed point, we also tried penalizing large absolute values of the time derivative *d***r**/*dt* in the window [*t*_1_, *t*_2_]. However, we found empirically that the penalty Eq (32) was sufficient to allow the training algorithms to find parameters for which the network converged with high probability to a fixed point.

### Goodness of fit analysis

We looked at the goodness of fit of model outputs by comparing the distributions of several scalar functions or “summary statistics” of tuning curves. We give the precise definitions of these statistics here.

For the feedforward network model of M1 tuning curves, in Experiment 1 we compared the data and model histograms of four test statistics or measures characterizing the hand-location tuning curves, defined as follows. Let $\bar{r}\left(s\right)$ denote the tuning curve, *i*.*e*., the trial average firing rate in condition *s*, with *s* ∈ {1, ⋯, 27} indexing the hand location from among the 3 × 3 × 3 cubic grid of target locations in the experiment of Ref. [4]). The average firing rate was simply $\frac{1}{27}\sum_{s=1}^{27}\bar{r}\left(s\right)$. The coding level of a tuning curve was defined as

$$\begin{array}{c} \text{Coding}\;\text{Level}=\frac{1}{27}\sum_{s=1}^{27}\Theta \left(\bar{r}\left(s\right)-5\;\text{Hz}\right) \end{array}$$ (34)

*i*.*e*., the fraction of conditions with rate larger than 5 Hz (Θ(⋅) denotes the Heaviside function). The *R*^2^ denoted the coefficient of determination of the optimal linear fit to the tuning curve, *i*.*e*.,

$$\begin{array}{c} R^{2}=1-\frac{\sum_{s=1}^{27}{\left(\bar{r}\left(s\right)-L\left(s\right)\right)}^{2}}{\sum_{s=1}^{27}\bar{r}{\left(s\right)}^{2}} \end{array}$$ (35)

where *L*(*s*) = *r*_0_ + **m** ⋅ **x**_*s*_ is the optimal linear approximation to $\bar{r}\left(s\right)$ (where *r*_0_ and **m** are the coefficients of the linear regression). Finally, following Ref. [4], the complexity score of a tuning curve was defined as in Eq (36).

$$\begin{array}{c} \text{Complexity}\;\text{Score}=\text{SD}\;[\frac{|\bar{r}\left(s\right)-\bar{r}\left(s^{\prime }\right)|}{\max_{s}\left(\bar{r}\left(s\right)\right)-\min_{s}\left(\bar{r}\left(s\right)\right)}|\;\left|x_{s}-x_{s^{\prime }}\right|=1] \end{array}$$ (36)

where SD denotes standard deviations.

To quantify the goodness of fit between the outputs of the ground truth and trained SSN models in Experiment 2, we compared the distributions of four test statistics characterizing the size tuning curves: preferred stimulus size, maximum firing rate, the suppression index, and the normalized participation ration, as defined below. While to fit the model we used size tuning curves containing responses to stimuli with *S* = 8 different sizes, *b*_*s*_, in the set (0, 1/16, 1/8, 3/16, 1/4, 1/2, 3/4, 1), for testing purposes and to evaluate the above measures, we generated tuning curves from the trained SSN using a larger set of stimulus sizes (denoted by *b* below). Letting $\bar{r}\left(b\right)$ denote the size tuning curve (*i*.*e*., $\bar{r}\left(b\right)$ is the sustained response of the center excitatory neuron to the stimulus with size *b*), the maximum firing rate is max_*b*_ $\bar{r}\left(b\right)$, and the preferred size is arg max_*b*_ $\bar{r}\left(b\right)$. The suppression index is defined by

$$\begin{array}{c} \text{Suppression}\;\text{Index}=1-\frac{\bar{r}\left(\max\left(b\right)\right)}{\max_{b}\left(\bar{r}\left(b\right)\right)}, \end{array}$$

and measures the strength of surround suppression. Finally, the normalized participation ratio (related to the inverse participation ratio [59]) is defined by

$$\begin{array}{c} \text{Normalized}\;\text{Participation}\;\text{Ratio}=\frac{1}{n_{b}}\frac{{\left(\sum_{b}\bar{r}\left(b\right)\right)}^{2}}{\sum_{b}\bar{r}{\left(b\right)}^{2}} \end{array}$$ (37)

and measures the fraction of all tested sizes that elicited responses comparable to the maximum response.

### Discriminator networks

The most studied application of GANs is in producing highly structured, high-dimensional output such as images, videos, and audio. In those applications, mathematical structures such as translational symmetry in the data space (for images) is exploited to design complex and structured discriminators such as deep convolutional networks. It has also been noted that the discriminator network should be sufficiently powerful so that it is capable of fully capturing the data and model distributions [29, 43, 44]. In our application, the outputs of the generator are comparatively lower-dimensional objects, with less complex distributions. Furthermore, developing a new discriminator architecture exploiting mathematical structure in the tuning curve space such as metric and ordering in the stimulus space is beyond the scope of this paper. In this work we used relatively simple discriminator networks. Nevertheless care is needed in designing discriminators; a function D that is too simple can preclude the fitting of important aspects of the distribution. For example, if a linear function D were used in the WGAN approach it would result in a fit that matches only the average tuning curve between model and data, and ignores tuning curve variability altogether.

For the M1 feedforward model, we used a dense feedforward neural network as the discriminator D, with four hidden layers of 128 rectified linear units and a single linear readout unit in the final layer. The discriminator network weights were initialized to uniformly random weights with Glorot normalization [60]. We do not use any kind of normalization or parameter regularization other than the WGAN penalty term in Eq (4) (i.e. we set Penalty_*G*_(***θ***) to zero in this example).

For the SSN recurrent model, we used dense feedforward neural networks with four hidden layers and with layer normalization [61] as recommended for WGAN in Ref. [26]. The discriminator network used in the experiments of Figs 4 and 5 had 128 and 64 neurons in each hidden layer, respectively. In the training experiments underlying Fig 6, we used networks with 32 to 128 neurons in each layer; as indicated by the WGAN histogram, all choices consistently performed well. We note that simple dense feedforward neural networks without normalization do not work well for SSN due to numerical instability in long-running training. It was important, however, *not* to apply the layer normalization in the first (input) layer, as the mean and variance across stimulus parameters of the turning curves are valuable information for the discriminator which would be discarded by such a normalization. We also used weight decay [62] for all parameters to stabilize the learning. We initialized all neural biases to 0 and initialized all weights as independent standard normal random variables, except in the input layer. For the input layers, we used the same initialization as in the M1 feedforward model’s discriminator.

### Moment matching

To provide a benchmark for our proposed GAN-based method, we also fit the SSN using moment matching [63]. We define a generic moment matching loss as

$$\begin{array}{c} L_{0}\left(\theta \right)=\frac{1}{D}\sum_{d=1}^{D}[w_{1,d}\;{\left(m_{d}\left(\theta \right)-\mu_{d}\right)}^{2}+w_{2,d}\;{\left(s_{d}\left(\theta \right)-\sigma_{d}\right)}^{2}]. \end{array}$$

Here, *D* = *S* × *O*, where *S* is the total number of stimulus conditions and *O* the number of neurons whose firing rates are probed in the SSN, and *d* indexes the combination of stimulus condition and probe neuron. *m*_*d*_(***θ***) and *s*_*d*_(***θ***) are the mean and variance of response in combination *d*, across a mini-batch of 32 model-generated sample tuning curves, respectively, while *μ*_*d*_ and *σ*_*d*_ are the empirical mean and variance of this response, across the full training dataset of 2048 size tuning curves. The *w*_*i*,*d*_ are the weights given to each moment, and are hyperparameters of the moment matching method. We tried the following options

$$\begin{array}{ccc} \text{uniform}\;\text{scaling}: & & w_{1,d}=(\frac{1}{D}\sum_{c=1}^{D}\mu_{c}{)}^{-2},\;w_{2,d}=\ell w_{1,d}^{2}, \end{array}$$ (38)

$$\begin{array}{cccc} \text{element-wise}\;\text{scaling}: & & w_{1,d}=(\mu_{d}+\varepsilon {)}^{-2}, & w_{2,d}=\ell w_{1,d}^{2}, \end{array}$$ (39)

$$\begin{array}{cccc} \text{relative}\;\text{scaling}: & & w_{1,d}=(\mu_{d}+\varepsilon {)}^{-2}, & w_{2,d}=\ell (\sigma_{d}+\varepsilon {)}^{-2}, \end{array}$$ (40)

where *ℓ* controls weight of the variance with respect to the mean and *ε* = 10^−3^ is a regularization constant to avoid division by zero. In our preliminary experiments, we found that element-wise Eq (39) and relative Eq (40) scalings are better than uniform scaling Eq (38). Thus, we only used element-wise Eq (39) and relative Eq (40) scalings in results presented here. For fitting the SSN, the same reasons for encouraging dynamical stability as in the WGAN case hold. Thus, we added the penalty term as defined in Eq (32) and minimized the loss

$$\begin{array}{c} L\left(\theta \right)=L_{0}\left(\theta \right)+{\text{Penalty}}_{G}\left(\theta \right). \end{array}$$ (41)

For most of the trainings, large firing rates yielding Penalty_*G*_(***θ***) > 0 rarely occured. The generator parameters are updated using Adam (a variant of stochastic gradient descent) with the hyperparameters *β*_1_ = 0.5, *β*_2_ = 0.9, *ϵ* = 10^−8^ as in Algorithm 1. We use the learning rate 0.001 unless specified.

### Stopping criterion and performance metric

To compare learned results between the GAN and moment matching and across different hyperparameters on an equal footing, we defined a condition for terminating learning as follows. First, we stop training at the first generator update step, *n*_0_, in which the speed-of-change of the generator parameters, as evaluated by

$$\begin{array}{c} \|\frac{1}{\nu }\sum_{n=n_{0}-\nu +1}^{n_{0}}\frac{\theta (n)-\theta (n-\kappa )}{\kappa }{\|}_{1} \end{array}$$ (42)

becomes smaller than a tolerance threshold of 0.01. Here ***θ***(*n*) is the vector of generator parameters at the *n*^th^ generator update, *κ* controls the timescale at which the speed is computed, and *ν* is the size of the moving average window. ‖**x**‖_1_ denotes the *L*_1_-norm of the vector **x**, i.e., the sum of absolute values of its components; thus the condition Eq (42) ensures that the speed-of-change of all model parameters are small, i.e., they have approximately converged. To obtain the final result (estimated parameters) of learning, we then compute the average of the generator parameter ***θ***(*n*) in the *ω* steps leading to step *n*_0_

$$\begin{array}{c} \hat{\theta }=\frac{1}{\omega }\sum_{n=n_{0}-\omega +1}^{n_{0}}\theta \left(n\right). \end{array}$$ (43)

For comparing results across different generator learning rates, *α*_*G*_, we used $\kappa =\nu =\omega =\alpha_{G}^{-1}$.

As the metric of performance, we use the so-called symmetric mean average percent error (sMAPE) of the estimated generator parameters $\hat{\theta }$ relative to the ground truth parameters ***θ***^truth^ which were used to generate the training dataset. That is we let

$$\begin{array}{c} \text{sMAPE}\left(\theta \right)=100\%\;\frac{1}{13}\sum_{i=1}^{13}\frac{|{\hat{\theta }}_{i}-\theta_{i}^{\text{truth}}|}{|{\hat{\theta }}_{i}+\theta_{i}^{\text{truth}}|/2} \end{array}$$ (44)

where 13 is the number of parameters. The performance in any learning run is then computed by the $\text{sMAPE}(\hat{\theta })$ of the final result, Eq (43), of that run obtained using the above termination procedure Eq (42). Note that this termination criterion is used only for Fig 6 and not in Fig 5, where we plotted the learning curve over a broader range for demonstration purposes.

### Hyperparameters for parameter identification experiment

For the WGAN-based fits shown in Fig 6 we picked combinations of hyperparameters from the following choices: the generator learning rate was *α*_*G*_ = 10^−4^, 2 × 10^−4^, or 4 × 10^−4^, the number of neurons in each discriminator hidden layer was 32, 64 or 128, discriminator update rule was Adam or RMSprop. For the moment matching fits the hyperparameter choices were: the learning rate was 10^−4^, 2 × 10^−4^, 4 × 10^−4^, 10^−3^, 2 × 10^−3^, or 4 × 10^−3^, the weight of variance λ = 0.01, 0.1, 1, and the moment scaling was element-wise Eq (39) or relative Eq (40).

### Implementation

We implemented our GAN-based method and moment matching in Python using Theano [64] and Lasagne [65]. Our implementation is available from https://github.com/ahmadianlab/tc-gan under the MIT license.
